# Evolution and biological characteristics of H11 avian influenza viruses isolated from migratory birds and pigeons

**DOI:** 10.1080/22221751.2024.2398641

**Published:** 2024-09-09

**Authors:** Jinyan Shen, Hong Zhang, Xiaohong Sun, Yaping Zhang, Mengjing Wang, Mengdi Guan, Lili Liu, Wenxi Li, Hongke Xu, Yujiao Xie, Anran Ren, Fengyang Cao, Wenqiang Liu, Guohua Deng, Jing Guo, Xuyong Li

**Affiliations:** a College of Agriculture and Biology, Liaocheng University, Liaocheng, People’s Republic of China; b Harbin Veterinary Research Institute of Chinese Academy of Agricultural Sciences, State Key Laboratory for Animal Disease Control and Prevention, Harbin, People’s Republic of China

**Keywords:** Avian influenza viruses, H11, migratory birds, epidemiology, poultry

## Abstract

The emergence of novel avian influenza reassortants in wild birds in recent years is a public health concern. However, the viruses that circulate in migratory birds are not fully understood. In this study, we summarized and categorized global H11 avian influenza viruses and reported that waterfowl and shorebirds are the major reservoirs of the identified H11 viruses. The surveillance data of the 35,749 faecal samples collected from wild bird habitats in eastern China over the past seven years revealed a low prevalence of H11 viruses in birds, with a positive rate of 0.067% (24 isolates). The phylogenetic analysis of the twenty viruses indicated that H11 viruses have undergone complex reassortment with viruses circulating in waterfowl and shorebirds. These tested viruses do not acquire mammalian adaptive mutations in their genomes and preferentially bind to avian-type receptors. Experimental infection studies demonstrated that the two tested H11N9 viruses of wild bird origin replicated and transmitted more efficiently in ducks than in chickens, whereas the pigeon H11N2 virus isolated from a live poultry market was more adapted to replicate in chickens than in ducks. In addition, some H11 isolates replicated efficiently in mice and caused body weight loss but were not lethal. Our study revealed the role of waterfowl and shorebirds in the ecology and evolution of H11 viruses and the potential risk of introducing circulating H11 viruses into ducks or chickens, further emphasizing the importance of avian influenza surveillance at the interface of migratory birds and poultry.

## Introduction

Influenza A viruses, members of the Orthomyxoviridae family, have a single-stranded negative RNA genome composed of eight segments [1]. They are divided into subtypes based on their two surface glycoproteins: haemagglutinin (HA) and neuraminidase (NA). To date, 18 HA subtypes and 11 NA subtypes have been detected in birds and mammals [2–4]. Recently, a novel HA subtype, designated H19, was discovered in common pochards in Kazakhstan and lesser scaups in northern California, USA [5, 6]. Influenza A viruses are characterized by their ability to infect a wide range of hosts, including humans, wild and domestic birds, pigs, horses, dogs and other terrestrial and marine mammals, making them of particular global public health concern [2].

Avian influenza viruses are a group of influenza A viruses that are highly diverse in birds, particularly in their waterfowl and shorebird reservoirs [7]. While avian influenza viruses circulate primarily in bird populations, their occasional spillover to mammals or humans has posed a substantial threat to public health because of the potential for pandemic emergence [8]. The global dissemination of highly pathogenic H5N1 viruses and their various reassortants, including H5N6 and H5N8 viruses, has had a devastating impact on wild bird populations and the poultry industry [9–12]. Increasing reports of H5N1 virus infections in humans and other mammals further illustrate the pandemic potential of zoonotic influenza [13–16]. In addition, the emergence of human infections with low-pathogenicity viruses in the last decade, such as the 2013 H7N9 [17, 18], 2014 H10N8 [19], 2018 H7N4 [20], 2021 H10N3, and 2022 H3N8 viruses [21–25], highlights that the spillover of viruses that are predominantly found in wild migratory birds at the human‒wildlife interface could trigger potential pandemics.

H11 influenza viruses were first identified in 1956 and have been found in migratory birds, chickens, ducks, geese, penguins, and pigs. As one of the rare subtypes, H11 viruses circulate mainly in waterfowl and shorebirds, and few H11 virus isolates have been identified in poultry [26]. In Asia, H11N6, H11N7, and H11N9 viruses have been reported in free-ranging ducks in Thailand, and the emergence of new reassortants has been observed [27]. As a unique lineage of H11 viruses, H11N2 influenza viruses were first detected in penguins in Antarctica in 2013, and their genome segments were found to be significantly different from those of contemporary influenza viruses [28, 29]. H11N2 viruses were also detected in environmental samples from Penguin Island in Antarctica in 2020 [30]. Despite previous reports on the surveillance of H11 viruses and information on more than 1000 H11 viruses, the monitoring of H11 viruses in different hosts, as well as evolutionary studies on circulating viruses, are particularly limited.

The binding of the influenza virus HA protein to sialic acid receptors on the host cell surface is the first step in initiating virus infection [31]. The α−2,3 and α−2,6 sialic acids are generally considered the most important host factors influencing the replication and transmission of avian influenza viruses [32]. Previous studies have reported that H11N2 viruses isolated from domestic birds at a Colombian live animal market in South America have an α−2,3-sialic acid binding preference and can replicate and transmit effectively in chickens [33]. Antarctic H11N2 penguin viruses have been shown to bind to avian-like receptors with limited replication in ferrets [28]. Moreover, an H11N3 virus of Eurasian origin has been isolated from environmental samples from a live poultry market in southeastern China, and animal studies have shown that the virus can replicate in mice and is capable of respiratory transmission in guinea pigs, although no binding affinity for human-type receptors was observed [34]. Although no clinical case of human infection has been reported, previous serological evidence has indicated infection with H11 viruses in humans with a long history of exposure to chickens, wild waterfowl and game birds [35, 36]. Because H11 viruses are predominantly found in wild birds and have rarely been identified in poultry or mammals, identification of their receptor binding specificity and evaluations of the replication and transmissibility of the circulating viruses in animal models are critical to clarify their spillover risks and pandemic potential.

Annual surveillance to monitor the circulation of avian influenza viruses in migratory birds has been initiated in several migratory bird habitats in Shandong Province, eastern China, since 2017. Here, we retrieved the surveillance results for the past seven years and analysed 20 H11 isolates to reveal their ecology, prevalence, evolution, and replication and transmission characteristics in animals. These findings will further fill the knowledge gap concerning H11 viruses and contribute to the surveillance of avian influenza viruses in migratory birds.

## Results

### Prevalence of global H11 influenza viruses in animals

**Figure 1.**
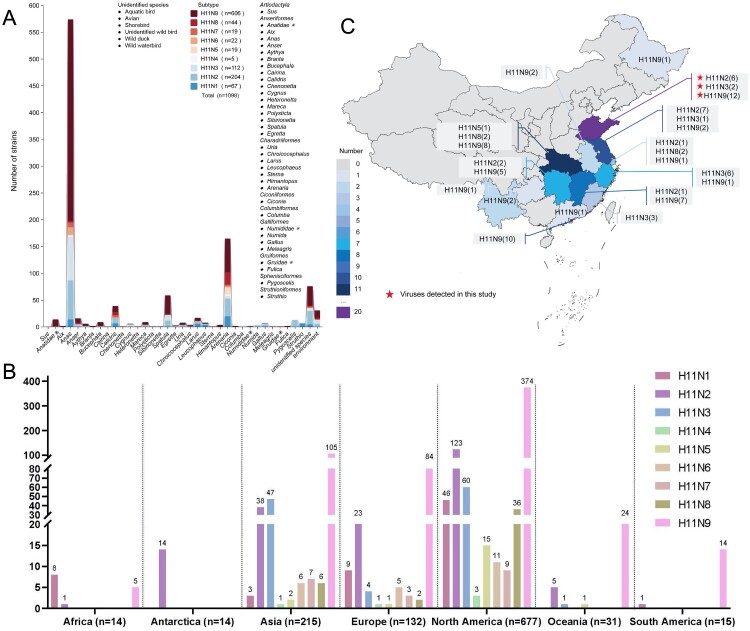
Hosts and geographical distributions of the global H11 influenza viruses. (A) H11Nx (N1 – N9) viruses in birds and mammals. The genera of the hosts are listed, and each genus contained at least one species. “*” indicates the family of the hosts. (B) Geographical distributions of the H11Nx (N1 – N9) viruses. (C) Number and distribution of the identified H11 viruses in China. The virus information and sequences are available from the GISAID and GenBank databases. All the data from the influenza virus databases were updated to 31 January 2024.

Ecological and epidemiological studies on avian influenza viruses have contributed significantly to active virus surveillance in different avian or mammalian populations [37, 38]. We summarized and categorized the host species and geographical distribution of available H11 strains from the GISAID and GenBank databases to better understand the epidemiological situation of H11 viruses (Figure 1, Table S1). To date (the data were updated to 31 January 2024), a total of 1098 H11 viruses, including nine HA (H11) and NA (N1–N9) subtype combinations, have been detected in their hosts. Notably, the H11N9 (*n* = 606), H11N2 (*n* = 204), and H11N3 (*n* = 112) viruses were the predominant subtype combinations (Figure 1(A)). These known H11 viruses have been identified in at least 71 bird species belonging to 31 genera of eight orders (Figure 1(A), Figure S1). Birds of the genus *Anas* are the primary reservoirs, as more than 52.28% (*n* = 574) of the known H11 viruses were identified in wild or domestic ducks, such as mallards (*Anas platyrhynchos*) (*n* = 338), common teals (*Anas crecca*) (*n* = 42), and domestic ducks (*n* = 136) (Figure 1(A), Figure S1). The birds of *Charadriiformes* are another important reservoir of H11 viruses, as almost 18.85% (*n* = 207) of the identified H11 viruses were detected in shorebirds, including ruddy turnstone (*Arenaria interpres*) and gulls (*Larus*) (Figure 1(A), Figure S1). A further detailed analysis revealed that birds of the order *Anseriformes* are the main reservoirs of the globally identified H11N2, H11N3, H11N6, H11N7, and H11N9 viruses, whereas the H11N1, H11N4, H11N5, and H11N8 viruses are predominantly restricted to birds of the *Charadriiformes* order (Figure S1). Interestingly, unlike avian influenza viruses, which are common in domestic birds, such as the H5, H7, and H9 viruses, spillover of H11 viruses from waterfowl or shorebirds to chickens and other terrestrial birds has rarely been reported, with only seven chicken H11 viruses found in databases to date. Except for a mammalian strain detected in pigs in South Korea in 2001, these global H11 viruses have not been found in humans or other mammals.

The geographical distribution of the H11 viruses revealed that the viruses were detected on seven continents worldwide. The North American lineage (*n* = 677) accounted for the majority of the identified H11 viruses, and 31.60% (*n* = 347) of the viruses were detected in Eurasia. Interestingly, H11N2 viruses were detected in penguins and environmental samples from Antarctica in 2013, as reported previously (Figure 1(B)) [28]. In China, the H11 virus was first discovered in ducks in Jiangsu Province in 2002. Over the past two decades, a total of 87 H11 viruses (including the 20 viruses in this study), including H11N2 (*n* = 17), H11N3 (*n* = 12), H11N5 (*n* = 1), H11N8 [4], and H11N9 (*n* = 52), have been identified in birds and environmental samples. Most of these H11 viruses were detected in waterfowl and shorebirds, and only two isolates (H11N2) were detected in chickens and one isolate (H11N2 in this study) was detected in pigeons (Figure 1(C), Table S2). Taken together, these detailed data indicate that waterfowl and shorebirds are the major reservoirs of the globally circulating H11 viruses.

### Isolation of H11 viruses from migratory birds and pigeons in eastern China

The wetlands located in Shandong Province of eastern China, including the Yellow River Delta and Swan Lake wetlands, are important habitats for migratory birds in the East Asian – Australasian (EAA) migratory flyway. Over the past seven years, our team has actively monitored avian influenza in migratory birds in several habitats in Shandong Province (Figure S2). Several subtypes, such as H3N8, H5N8, H9N2, H10N4, and H10N8, have been detected successively in shorebirds and waterfowl and were characterized in our previous studies [11, 39–43]. From September 2017 to December 2023, the Yellow River Delta wetland and Swan Lake wetland in Shandong Province were monitored on 51 separate occasions, and 35,749 wild bird samples were collected. Overall, 319 avian influenza viruses of 14 HA subtypes were isolated from the samples, with an isolation rate of 0.89%. Of these, 24 H11 viruses were recovered from migratory birds (Table 1). In addition, an H11N9 virus was isolated from 560 egret samples in the Tuhai River wetland in 2022 (Table S3, Figure S2). Notably, the H11 virus was first isolated from egrets, which are common aquatic birds. In addition, the pigeon H11N2 virus was first detected in a live poultry market in 2019 (Table S3, Figure S2). These epidemiological surveillance data and the isolation results indicate that H11 viruses are present in different species of birds in the EAA flyway, although the percentage of H11-positive birds was low (0.067% in migratory birds).

**Table 1.** Description of sampling of migratory wild birds for detection of avian influenza viruses at two major wild birds habitats in Shandong province of eastern China, September 2017–December 2023 (*n* = 35,749 samples collected).

Annual period	Location*	Number of samples collected	Times of surveillance	Number of H11 isolates	HA subtypes (number) of AIV isolates**	Isolation rate (%)
Sep–Dec, 2017	a	937	4	0	H3 (6), H4 (1), H5 (3), H6 (4)	1.49
	b	607	1	0	H1 (3), H2 (2)	0.82
Jan–Dec, 2018	a	1887	2	5	H4 (1), H6 (3), H7 (3)	0.64
Jan–Dec, 2019	a	7679	7	5	H1 (7), H3 (16), H4 (12), H5 (2), H6 (31), H9 (12), H12 (1)	1.12
	b	2574	5	1	H4 (1), H5 (1), H7 (15), H9 (2)	0.82
Jan–Dec, 2020	a	4581	8	1	H1 (2), H2 (1), H3 (4), H5 (8), H6 (3), H7 (4), H10 (2)	0.55
	b	612	1	0	H1 (1), H3 (3), H4 (1), H5 (1), H10 (5)	1.8
Jan–Dec, 2021	a	2692	4	0	H2 (1), H3 (4), H5 (5), H6 (3), H9 (1), H10 (2), H13 (7), H16 (1)	0.89
	b	1032	4	3	H2 (1), H5 (18), H10 (3)	2.42
Jan–Dec, 2022	a	4943	5	0	H1 (2), H2 (1), H3 (8), H5 (1), H7 (1), H8 (1), H13 (7)	0.45
	b	823	2	3	H5 (7)	1.22
Jan–Dec, 2023	a	6205	6	2	H1 (3), H3 (2), H4 (9), H5 (13), H6 (3), H7 (9), H9 (8), H10 (4), H13 (3), H16 (3)	0.95
	b	1177	2	4	H4 (3)	0.59
Total		35,749	51	24	13 HA subtypes (295)	0.89

*a: Yellow River Delta wetland, Shandong, China; b: Swan Lake wetland, Shandong, China.

**All the HA subtypes, excluding the H11 subtype, and the number of avian influenza viruses isolated from the wild birds samples.

### Genetic and phylogenetic analyses of the H11 viruses

**Figure 2.**
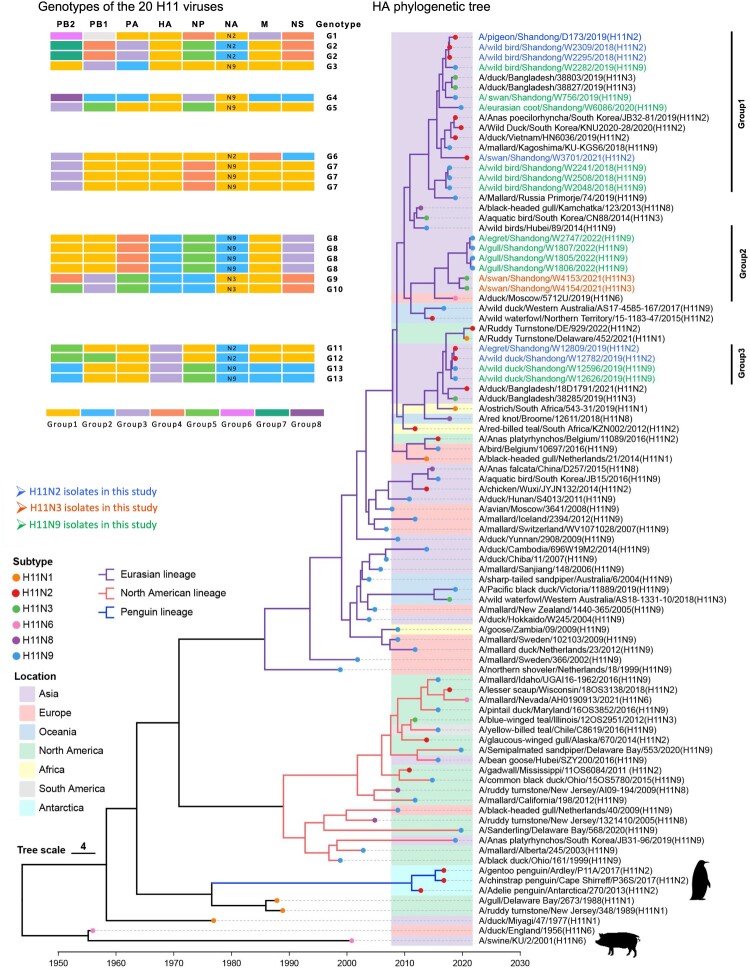
Phylogenetic analysis of H11 viral haemagglutinin nucleotide sequences from different hosts and regions of the world, including 20 sequences retrieved in this study from 2018 to 2022 (*n* = 91 sequences in total).

Six H11N2, two H11N3, and twelve H11N9 viruses that were detected from 2017 to 2022 were further analysed to understand the evolutionary relationships of the H11 viruses (Table S3). Nineteen of these H11 viruses were isolated from 6408 faecal droppings of waterfowl (*Cygnus cygnus*, unidentified wild ducks, and *Fulica atra*), shorebirds (*Larus* and *Egretta*), and other unidentified migratory birds from three wetlands in Shandong Province. The pigeon H11N2 virus was also analysed to compare its genetic differences with those of wild bird-origin viruses (Table S3). The genomes of the 20 viruses were sequenced and compared with those of the available strains reported by others (Table S4). The time-scaled phylogenetic tree of HA of the H11 viruses was inferred from the HA sequences of the 91 H11 viruses, including the 20 H11 viruses sequenced in this study and 71 HA sequences downloaded from GISAID and GenBank, according to their HA and NA subtype combinations, locations and host species (Figure 2). Overall, most of the HA genes of the H11 viruses clustered into the Eurasian lineage and North American lineage, whereas a few isolates belonged to the penguin lineage, which descended from the old lineage of American wild bird viruses. The phylogenetic tree shows that the HA genes of the H11 viruses have undergone complex changes over time. The Eurasian lineage was formed mainly by the viruses detected in migratory birds and ducks in Asia, Europe, Oceania, and Africa, while the North American isolates in this lineage suggested that Eurasian H11 viruses were introduced into North America. The viruses detected in China and South Korea suggested that North American lineage viruses can also be transmitted to Eurasia. The penguin H11N2 viruses form a unique lineage and have been detected mainly in penguins in Antarctica [44]. The 20 viruses characterized in this study clustered into the Eurasian lineage and shared high genetic similarity with H11 viruses circulating in Asia, such as those in South Korea, Japan, Russia, and Bangladesh, which are located in the EAA migratory flyway. The HA genes of the 20 viruses presented 92.4−100% genetic similarity and could be divided into three groups (Figure 2).

**Figure 3.**
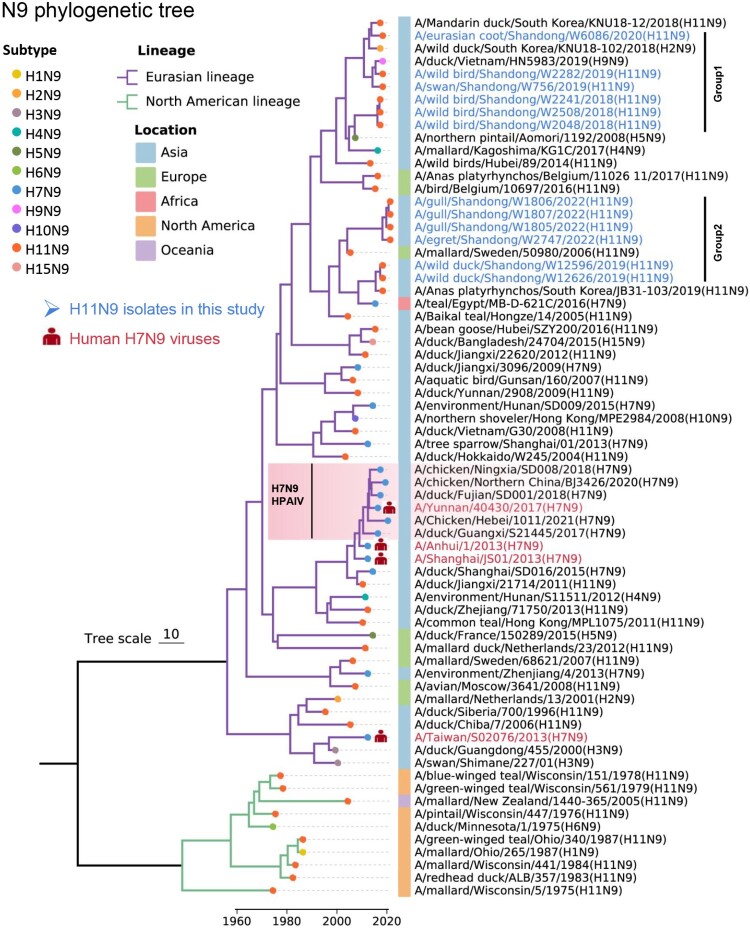
Phylogenetic tree of neuraminidase (N9) genes from birds and mammals worldwide, including 12 H11N9 viruses isolated in this study (*n* = 69 sequences in total).

Time-scaled phylogenetic trees of the NA genes were generated to understand the phylogenetic characteristics and genetic relationships of the NA genes (N2, N3, and N9) of the twenty H11 viruses isolated in this study. The phylogenetic tree of the N2 genes, including six sequences in this study and 48 sequences from GISAID and GenBank, revealed the evolution of the NA genes by reassortment with the viruses circulating in ducks and wild waterfowl. The NA genes of the six H11N2 viruses shared 95.8%−100% nucleotide similarity and clustered into two groups with the viruses detected in China and other Asian countries (Figure S3). The NA gene sequences of the two swan H11N3 viruses isolated in this study and 63 HxN3 viruses downloaded from GISAID and GenBank were used to generate a phylogenetic tree and reveal the genetic relationship of the N3 genes. The NA genes of the two swan H11N3 viruses shared 100% nucleotide identity. The phylogenetic tree indicates that the N3 genes of the viruses evolved in the Eurasian lineage and North American lineage. The N3 gene can bind to different subtypes of HA, and HxN3 viruses circulate mainly in waterfowl and shorebirds, although Eurasian lineage H7N3 viruses have been detected in chickens. The two swan H11N3 viruses isolated in this study shared high nucleotide identity with the H1N3, H5N3, and H10N3 viruses detected in samples from ducks or unidentified wild birds in East Asia (Figure S3). The N9 phylogenetic tree was inferred using the 57 NA sequences of HxN9 viruses from databases and the twelve H11N9 viruses isolated in this study. Obviously, the Eurasian lineage N9 genes have undergone more evolutionary occurrences and generated more complex reassortants than the North American lineage genes. The spillover of the NA gene from wild birds to chickens and humans through the emergence of H7N9/2013 reassortants facilitated their evolution and genetic divergence (Figure 3). The twelve H11N9 viruses isolated in this study shared 93.5−100% nucleotide identity and high genetic similarity with viruses detected in unidentified wild birds and ducks in Eurasia (Figure 3).

The six internal genes of these twenty viruses, namely, PB2, PB1, PA, NP, M, and NS, shared 88−100%, 91.1−100%, 91.1−100%, 90.5−100%, 91−100%, and 70.5−100% nucleotide identity, respectively. The phylogenetic analysis of the viruses isolated in this study revealed the evolutionary divergence of the internal genes and the complex reassortment with viruses of duck or wild bird origin detected in Eurasia (Figure S4). The PB2 segments were the most diverse genes because eight groups were divided according to the phylogenetic tree and their genetic differences (the nucleotide identity between each group of gene segments was lower than 95.9%). The phylogenetic analysis revealed that the PB1 genes of the H11 viruses in Groups 1 and 2 were similar to those of the H5 viruses that circulate in birds. The M gene of the D173 virus formed a unique group with H5N6 viruses that have been detected in birds and mammals, suggesting that this virus is a reassortant that originated from wild and domestic birds (Figure S4). Importantly, the internal genes of some of the H11 isolates used in this study are highly homologous to the genes of viruses that have been identified in migratory birds in the same sampling regions, as previously reported [11, 39–43], suggesting that the internal genes of these H11 viruses originated from the gene pools of the viruses that are mainly found in waterfowl and shorebirds in the EAA flyway and further act as gene donors for the emerged viruses (Figure S4). The twenty viruses retrieved in this study were grouped into 13 genotypes based on the phylogenetic diversity of each gene segment and the groups of each gene segment (≥95.9 nucleotide identity in one group) (Figure 2). These genetic and phylogenetic results indicate the complex reassortment and evolution of H11 viruses in ducks and other migratory birds [45].

### Molecular characteristics of the H11 viruses

The conserved amino acid motif IASR. GLF at the HA cleavage site suggested the low pathogenicity of the H11 viruses in chickens. No amino acid mutation in the receptor binding domain of HA was observed to increase the receptor binding specificity to the human-type receptor (Table S5). The glutamic acid (E) and aspartic acid (D) at positions 627 and 701 in PB2 indicate that the H11 isolates have not acquired the typical mammalian adaptation in their polymerase subunits (Table S5).

### Receptor binding specificity of the H11 isolates

**Figure 4.**
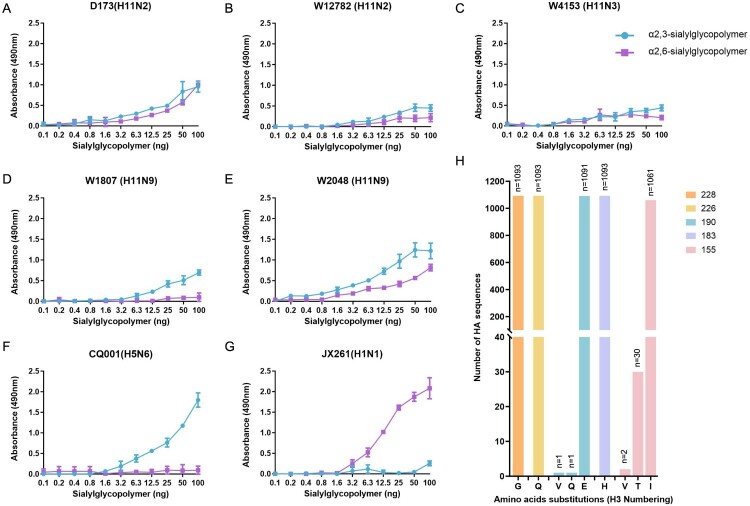
Receptor binding specificity of the representative H11 viruses used in this study and analysis of the amino acid substitutions in the receptor binding domain in the HA proteins of all the available H11 viruses (*n* = 1093 in total). (A-E) Receptor binding specificity of five H11 viruses to SA α-2,3-sialylglycopoylmer and SA α-2,6-sialylglycopoylmer. (F and G) Receptor binding specificity of the control viruses (CQ001 (H5N6) and JX261 (H1N1)). (H) Amino acid substitutions at positions 155, 183, 190, 226, and 228 in the HA proteins of the global H11 viruses. The full-length sequences of HA were downloaded from GISAID and GenBank. The sequence data of H11 viruses in GISAID and GenBank used in this study were up to date as of 31 January 2024.

Receptor binding specificity is a prerequisite for cross-species infection and transmission of avian influenza viruses from birds to mammals. Two H11N2 viruses, one H11N3 virus, and two H11N9 viruses were selected, and SA α−2,3-sialylglycopoylmer and SA α−2,6-sialylglycopoylmer were used to test their receptor binding properties. Although H11 viruses have acquired dual receptor binding properties, the binding specificity for the avian-type receptor (SA α−2,3-sialylglycopoylmer) of these tested viruses was greater than their affinity for the human-type receptor (SA α−2,6-sialylglycopoylmer) (Figure 4(A–E)). However, H11 viruses, especially W12782, W4153, and W1807, exhibited a lower binding ability to both avian – and human-type receptors than chicken H5N6 and swine H1N1 viruses did (Figure 4(A–G)). Key amino acid substitutions in the receptor binding domain of HA determine the receptor binding specificity of influenza viruses. We analysed the amino acids at positions 155, 183, 190, 226, and 228 in HA (H3 numbering) of all available H11 viruses in GenBank and GISAID, including the 20 viruses in this study (*n* = 1093 in total), to further elucidate the key amino acid substitutions in HA of H11 viruses. Only a few strains (*n* = 30) possessed 155 T, which facilitates virus binding to human-type receptors, while most of the isolates (*n* = 1061) (including 20 viruses in this study) possessed 155I in HA. Interestingly, 183H, 226Q and 228G are found in the HA proteins of all H11 strains (*n* = 1093), and 190E is found in the HA proteins of 1091 H11 viruses, which collectively contribute to the avian-type receptor binding property of avian influenza viruses (Figure 4(H)). These results indicate that naturally isolated avian H11 viruses have conserved receptor binding domains that support their preferential binding specificity to avian-type receptors.

### Replication and transmission of H11 viruses in ducks

**Figure 5.**
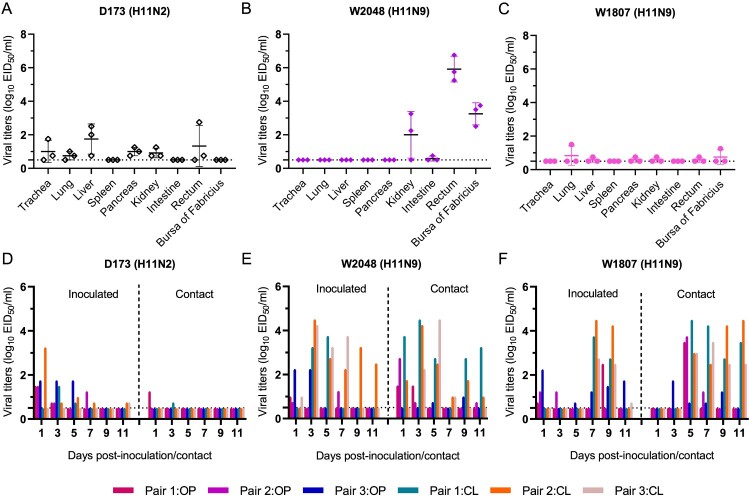
Replication and transmission of H11 viruses in ducks. One pigeon H11N2 virus and two H11N9 viruses detected from wild birds and gulls were tested in ducks. The viral titres of the organs, tissues and swabs were detected in eggs. The dashed line indicates the lower detection limit.

As more than 61.11% of the known H11 viruses have been detected in birds of the genus *Anas*, ducks may be the major reservoir of natural H11 viruses. However, few studies have reported experimental H11 virus infections in ducks. Therefore, here, we used specific-pathogen-free (SPF) ducks to study the infectivity and replication of H11 viruses. The properties of three viruses, namely, pigeon H11N2 virus (D173), wild bird H11N9 virus (W2048), and gull H11N9 virus (W1807), were tested in ducks. The virus titres in the organs or tissues indicated that the pigeon H11N2 virus (D173) can replicate in the trachea, lung, liver, pancreas, kidney, and rectum of the ducks, although the replication of the virus was maintained at a relatively low level (0.75–1.75 log_10_ 50% egg infections dose (EID_50_)/ml) (Figure 5(A)). The wild bird H11N9 virus (W2048) exhibited an efficient replication ability and was detected in the kidney, intestine, rectum and bursa of Fabricius, with viral titres ranging from 0.5 to 6.35 log_10_ EID_50_/ml. However, the virus could not be detected in trachea or lung samples, although viral shedding was detectable from the oropharyngeal swabs of the inoculated ducks (Figure 5(B, E)). The gull H11N9 virus (W1807) exhibited poor replication in ducks, as low viral titres (0.55–0.75 log_10_ EID_50_/ml) were detected in several organs or tissues of the birds (Figure 5(C)). We then tested the degree of viral shedding of the three viruses and their transmissibility in ducks. In the transmission study, the three viruses were detected in the oropharyngeal and cloacal swabs of inoculated ducks, and the viral shedding period of these viruses reached 11 dpi. Although shedding of the three tested viruses was detected in the inoculated ducks, their transmissibility was variable. The W2048 and W1807 viruses detected from unidentified wild birds and gulls were more transmissible than the pigeon D173 virus (Figure 5(D-F)). In the contact group, the D173 virus was detected in only one bird at 1 and 3 dpi, with low viral titres (1.25 and 0.75 log_10_ EID_50_/ml, respectively), whereas the viral titres of W2048 and W1807 in the contacted ducks reached 4.25 log_10_ EID_50_/ml (Figure 5(D–F)). In addition, these H11 viruses can induce the production of HI antibodies in the serum of both inoculated ducks and contact ducks (Figure S5(A-C)). These experimental infection studies indicate that naturally isolated H11 viruses, especially those originating from wild birds, pose a high risk of infection in domestic ducks.

### Replication and transmission of H11 viruses in chickens

**Figure 6.**
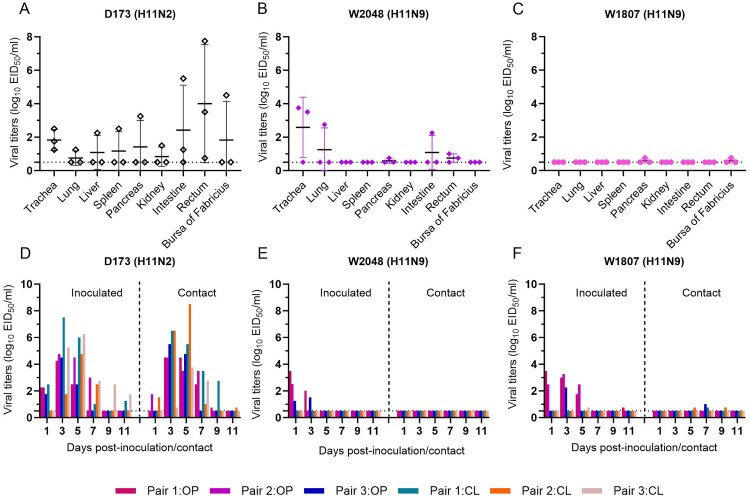
Replication and transmission of H11 viruses in chickens. Chickens were inoculated with the tested viruses, and viral titres were detected in eggs. The dashed line indicates the lower detection limit.

To date, only 7 of the total global H11 viruses (*n* = 1098) have been detected in chickens, and their infection and replication in chickens are not fully understood. The H11N2 and H11N9 viruses were further tested in SPF chickens to evaluate the risk of circulating H11 viruses being introduced into chickens. The D173 virus was detected in all the organ and tissue samples collected from the inoculated chickens, suggesting that the pigeon virus exhibited systemic replication in chickens. Viral shedding from the inoculated and contact chickens indicated that the pigeon H11N2 virus had adapted to replicate in chickens and could be transmitted efficiently between chickens (Figure 6(A,D)). In contrast to the pigeon virus, the wild bird W2048 virus exhibited lower replication in chickens, although the virus was detected in the trachea, lung, pancreas, intestine, and rectum of inoculated chickens, with viral titres ranging from 0.75 to 3.75 log_10_ EID_50_/ml. The virus was detected in oropharyngeal swabs at 1 and 3 dpi, with viral titres ranging from 1.25 to 3.5 log_10_ EID_50_/ml. No virus shedding was detected in cloacal swabs from inoculated chickens, and no virus was detected in the contact chickens (Figure 6(B,E)). The gull W1807 H11N9 virus replicated poorly in chickens, as the virus was detected in only two organs of the inoculated chickens, with viral titres of 0.75 log_10_ EID_50_/ml. The transmission study also suggested that this virus has not adapted to infect chickens (Figure 6(C,F)). The HI titres in the serum of both the inoculated and contact chickens indicated that the pigeon H11N2 virus can induce higher levels of HI antibodies than the two wild bird-origin H11N9 viruses can (Figure S5(D-F)). These infection studies in chickens implied that the H11 virus isolated from poultry markets poses a greater risk of infection in chickens than do the viruses circulating in wild migratory birds.

### Replication and virulence of H11 viruses in mice

**Figure 7.**
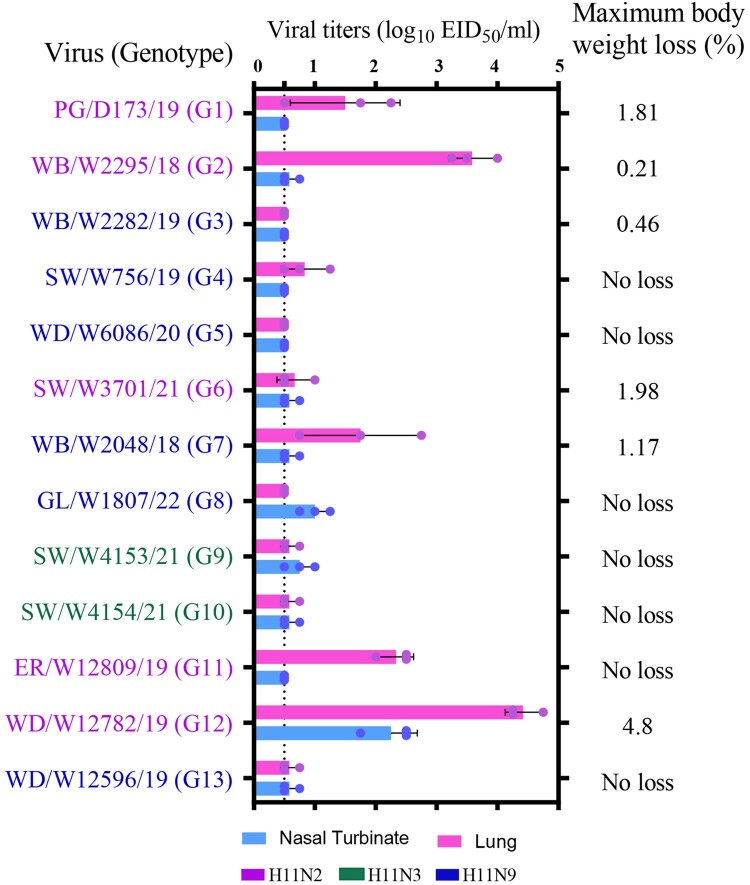
Replication and virulence of H11 isolates from Eastern China (*n* = 13 strains) in mice. At least one isolate was selected as the representative virus from each genotype. Mice were inoculated intranasally with 10^6^ EID_50_/ml in a volume of 50 μl, and the virus titres of the organs or tissue were measured in eggs. The change in body weight of the inoculated mice was monitored for two weeks. The dashed line indicates the lower detection limit.

Thirteen isolates, including five H11N2, two H11N3 and six H11N9 viruses from each genotype, were further studied in mice to assess the risk of infection and replication of H11 viruses of wild bird and pigeon origin in mammals. These representative viruses exhibited variable replication in the nasal turbinates or lungs of inoculated mice. Eleven of the 13 viruses were detected in the nasal turbinates or lungs of the mice, with viral titres ranging from 0.75 to 4.75 log_10_ EID_50_/ml, although several isolates replicated poorly (Figure 7). The viruses WB/W2295/18 and WD/W12782/19 replicated efficiently in mice, with viral titres of up to 4.0 and 4.75 log_10_ EID_50_/ml in the lung, respectively. No virus was detected in the kidney, spleen or brain of the mice (data not shown). Four H11N2 viruses and two H11N9 viruses caused 0.21% to 4.8% body weight loss in mice but were not lethal (Figure 7). These results indicate that the H11 viruses isolated from waterfowl, shorebirds and pigeons exhibit differential pathogenicity in mice and that some of these isolates are able to efficiently replicate in mice without prior adaptation, suggesting a potential risk of mammalian infection.

## Discussion

In this study, based on the surveillance data collected in the past seven years, H11 viruses circulating in migratory birds and pigeons in Shandong Province, eastern China, were fully characterized. The ecology, prevalence, evolution, and replication and transmission characteristics of the viruses revealed in this study will help us evaluate the infection risk of H11 viruses in different species.

Wildlife are considered natural reservoirs of pathogens with zoonotic potential [46, 47]. The spillover of avian influenza viruses from migratory birds to poultry or mammals has contributed to the emergence of novel reassortants or mutants. The emergence of H7N9, H7N4, H10N3, and H3N8 influenza viruses and their infections in humans in the last decade have led to concerns about the potential for pandemics [48-52]. Previous studies have confirmed that these viruses originate from wild bird viruses and poultry viruses [20, 53-55]. Unfortunately, all of these emerged viruses or reassortants were first identified in humans, not in poultry or wild birds. The active, long-term and systematic surveillance of avian influenza viruses in birds will help us monitor viruses that have potential infection and cross-species transmission risks to humans [56]. Over the past seven years, continuous surveillance for avian influenza viruses in migratory birds has been conducted in several important wetlands in Shandong Province, Eastern China. The cumulative surveillance results help us understand the ecology of avian influenza viruses in wild waterfowl and shorebirds and their infection risks in domestic birds and mammals.

Although wild birds and poultry are considered susceptible hosts for avian influenza viruses, the reservoirs of different subtypes of viruses differ significantly. A total of 35,749 faecal samples of wild migratory birds were collected from the YRD wetland and Swan Lake wetland over the past seven years to monitor the prevalence of avian influenza viruses, but only 319 viruses were successfully isolated and identified from the samples, which implies a low prevalence (0.89%) of avian influenza viruses in migratory birds in eastern China. Notably, a significant proportion of the viruses with haemagglutinating properties isolated from the 35,749 samples have not been successfully sequenced and identified, making the identification of these unknown viruses difficult. Deep sequencing and other next-generation sequencing methods will be used in our future studies to identify these unknown viruses. Environment samples (faeces, water, air, mud and swabs of surfaces) have been used effectively for avian influenza surveillance, despite this sampling method will not be sufficient to detect virus when the prevalence of infection or contamination is low compared with the live bird samples [57]. In this study, we collected 35,749 samples by sampling faeces but not directly swabbing live birds. Faecal sampling is more suitable for active avian influenza surveillance because many samples need to be collected due to the low positive rate of avian influenza viruses in wild migratory birds. Although the direct swabbing method has difficulty meeting the quantitative demands of sampling in active avian influenza surveillance, it is well suited for virus identification in sick or dead birds [58, 59]. In contrast to the viruses commonly found in chickens, such as the H5, H7, and H9 subtypes, H11 viruses are rarely detected in chickens. In this study, we found that H11 viruses were detected mainly in ducks (including domestic ducks), followed by shorebirds, such as ruddy turnstones (*Arenaria interpres*) and gulls (*Larus*). The host reservoir preference of the H11 viruses was similar to that of the H3N8 and H12N5 viruses of wild bird origin, which were detected mainly in ducks (including domestic ducks) and shorebirds [23, 39]. To date, only 1098 global H11 viruses, including the 87 viruses reported in China, have been deposited in GISAID and GenBank since the first isolate was detected in 1956. In this study, a total of 24 H11 viruses were isolated from 35,749 samples of wild migratory birds, with a positive rate of 0.067%, indicating a low prevalence of H11 viruses in migratory birds in eastern China.

Avian influenza viruses isolated from poultry, such as H7N9 and H9N2, have acquired essential amino acid mutations in the receptor binding domain to increase their binding specificity to human-type receptors and efficient transmission in ferrets [18, 60]. In this study, we found that the tested H11 viruses preferentially bound to the avian-type receptor, which was consistent with previous reports [28, 33, 34]. These tested viruses share similar receptor binding properties with the Antarctic H11N2 penguin viruses, the Colombian H11N2 viruses, and the H11N3 virus of wild bird origin detected in southeastern China [28, 33, 34]. Statistical analysis of amino acid substitutions in the receptor domain in the HA of all global H11 viruses further revealed the molecular basis for their receptor binding phenotype. Several factors, such as the specificity of the primary antibodies and the NA activity of the tested viruses, may affect the binding ability of the virus to sialic acid in the receptor binding assay. Compared with W2048 and the control viruses, three tested viruses (W12782, W4153, and W1807) did not efficiently bind to either the SA α−2,3-sialylglycopoylmer or the SA α−2,6-sialylglycopoylmer. More detailed experiments are needed to elucidate the determinants that influence the receptor binding ability of H11 viruses in future studies. SPF chickens and ducks have been widely used to evaluate the infectivity, pathogenicity and transmissibility of avian influenza viruses in domestic birds [61, 62]. In previous studies, we reported that the wild bird-origin H3N8, H8N4, H9N2, H10N4, and H10N8 viruses replicated and were transmitted more efficiently in ducks than in chickens, but the H12N5 and H16N3 viruses were poorly adapted to domestic ducks and chickens [23, 39–42, 63]. The avian infection experiments in this study further indicated that the two wild bird H11N9 viruses can be efficiently transmitted to ducks but not to chickens. Interestingly, the pigeon H11N2 virus isolated from a live poultry market showed poor transmissibility in ducks, although it could be detected in the organs of inoculated ducks. However, the pigeon H11N2 virus has adapted to replicate and transmit in chickens. The findings of this study suggest that the H11 viruses isolated from wild migratory birds and the live poultry market exhibited adaptation in ducks and chickens, and the molecular basis for this difference needs further investigation.

In conclusion, through surveillance data collected over the past seven years in eastern China and the available information from databases, we described the ecological landscape and genetic characteristics of H11 viruses. The findings concerning the biological properties of the circulating H11 viruses revealed their potential spillover into domestic birds and mammals. Long-term surveillance of avian influenza viruses in wild migratory birds and domestic poultry is necessary to better understand the characteristics of H11 avian influenza viruses and should be strengthened in the future.

## Materials and methods

### Ethics statement and experimental animals

The experimental protocols for chickens, ducks and mice were approved by the Ethics Committee for Animal Experimentation of Liaocheng University (ethics approval number: 2023022708). Six-week-old SPF chickens and three-week-old SPF ducks were purchased from Shandong Healthtech Laboratory Animal Breeding Co., Ltd. (Ji’nan, Shandong, China). Six-week-old SPF female BALB/c mice were obtained from Jinan Pengyue Experimental Animal Breeding Co., Ltd. (Ji’nan, Shandong, China).

### Sequence data acquisition from databases

HA sequences of all H11 viruses were downloaded from the Global Influenza Data Sharing Initiative (GISAID; https://www.gisaid.org) and the National Center for Biotechnology Information (NCBI; https://www.ncbi.nlm.nih.gov) databases and imported into MEGA 11 to remove overlapping sequences. Information on the host, isolation site, isolation time and virus subtype was statistically analysed. The number of sequences of H11 viruses in different hosts and subtypes was counted. Based on the virus isolation information, the intercontinental subtype distribution of H11 viruses and the spatial distribution of H11 viruses in China were determined. Public virus sequences available in the NCBI and GISAID databases were updated to 31 January 2024. The H11 virus data were analysed using GraphPad Prism 8.0.2.

### Surveillance sites and sample collection

The wetlands in Shandong Province, eastern China, which provide ideal habitats for the migratory birds of the East Asia – Australasia (EAA) flyway, were selected as the wild bird monitoring sites. The Yellow River Delta wetland is the largest wild migratory bird habitat in Shandong Province, whereas the Swan Lake wetland in Shandong Province is the major wintering habitat for whooper swans in eastern China. Annual viral surveillance of wetlands in Shandong Province began in 2017, and nearly all the samples were faecal droppings of migratory birds. The species of the wild migratory birds in the habitats were first determined using binoculars and then photographed by a telephoto lens or unmanned aerial vehicle (UAV) to identify the host species of the faecal droppings. If only one species was observed in a specific habitat, fresh faecal droppings could be linked to a particular bird. If several species were grouped together, the viruses isolated from faecal droppings were named from wild birds. Once an individual faecal sample was collected with a swab, its neighbouring droppings were discarded if they descended from the same bird individual. For the 19 wild migratory bird-originating viruses used in this study, 13 viruses were isolated from the faecal droppings of swans, wild ducks, gulls, egrets, and Eurasian coots, while the other six viruses were named from wild birds. The oropharyngeal (OP) and cloacal (CL) swabs of the pigeon samples included in this study were collected from live pigeons that were sold at a live poultry market in Shandong Province. OP and CL swabs and faecal samples were collected in 2 ml of PBS containing penicillin and streptomycin at 2–8°C for transportation and at −80°C for storage.

### Virus isolation and identification

The samples stored at −80 °C were first thawed, centrifuged at 12,000 rpm for 3 min, and then subjected to RT‒PCR using specific M and HA (H5 and H7) primers to determine whether they were positive for H5 or H7 avian influenza viruses [64, 65]. The highly pathogenic H5- or H7-positive samples identified by RT-PCR were filtered out and transferred to an ABSL-3 facility at the Harbin Veterinary Research Institute of the Chinese Academy of Agricultural Sciences for further virus identification and isolation. The remaining samples were injected into 10-day-old embryonated chicken eggs for virus isolation in an ABSL-2 laboratory at Liaocheng University.

### Molecular analysis

Viral RNA was extracted from the allantoic fluid of virus-infected eggs, and cDNA was synthesized via reverse transcription (RT) using the Uni12 primer 5′-AGCRAAAGCAGG-3′. PCR was performed for each of the eight genes using gene segment-specific primers (Table S6). The PCR products of each gene segment were then sequenced on an Applied Biosystems DNA analyzer (3500xL Genetic Analyzer, United States of America) according to the manufacturer’s instructions. Sequence data were compiled using the SeqMan programme (DNASTAR, Madison, WI, USA).

### Phylogenetic analysis

Phylogenetic analyses were performed using the sequences of the 20 viruses investigated in this study and the full-length HA sequences downloaded from NCBI and GISAID. Multiple sequence alignment was constructed using MAFFT software (V7.505) with the default settings. The time-resolved tree was inferred using a time-measured Bayesian Markov chain Monte Carlo (MCMC) analysis with BEAST software (v1.10.4). The best-fitting nucleotide substitution model was selected using IQ-tree (v2.2.0). Tracer (v1.7.1) was used to check for effective sample sizes (ESSs) greater than 200. After a burn-in of 10%, the maximum clade credibility (MCC) tree was generated for each dataset using Tree Annotator in BEAST. The ML trees of the six internal genes (PB2, PB1, PA, NP, M, and NS) were obtained using IQ-tree, and their robustness was determined through an ultrafast bootstrap resampling analysis of 1000 replicates. The Tree Visualization By One Table (tvBOT) online service (https://www.chiplot.online/tvbot.html) was used to visualize and beautify the trees [66].

### Receptor binding assay

The receptor binding specificity of the H11 viruses was assessed using a solid-phase receptor binding assay, as described previously [42, 63]. Briefly, the synthetic glycopolymers α2,3-sialylglycopolymer [Neu5Ac2-3Galβ1-4GlcNAcβ1-pAP (para-aminophenyl)-alpha-polyglutamic acid (α-PGA)] and α2,6-sialylglycopolymer [Neu5Ac2-6Galβ1-4GlcNAcβ1-pAP (para-aminophenyl)-alpha-polyglutamic acid (α-PGA)] were coated in 96-well plates to bind to the purified viruses. The chicken polyclonal antibody specific for HA of H11 viruses was generated previously by immunization with the expression plasmid encoding PCAGGs-HA of the H11N9 virus WB/W2048/18 in SPF chickens, and its HI antibody titre against the tested H11 viruses ranged from 128 to 256. The HA antibody was diluted 300-fold in PBS and then reacted with the bound viruses. Dose‒response curves of virus binding to the glycopolymers were analysed using a single-site binding algorithm and curve fitting with GraphPad Prism 9. Each value is presented as the mean ± standard deviation (SD) of three independent experiments, each performed in triplicate. The chicken H5N6 virus A/chicken/Chongqing/SD001/2021(H5N6) (CQ001 (H5N6)), which binds primarily to avian-type receptors, and the swine H1N1 virus A/swine/Jiangxi/261/2016 (H1N1) (JX261 (H1N1)), which binds predominantly to human-type receptors, were used as a pair of controls in this study.

### Duck and chicken infection studies

Six six-week-old SPF chickens or six three-week-old SPF ducks seronegative for avian influenza viruses were inoculated intranasally (i.n.) with 0.1 ml of 10^6^ EID_50_ of the tested viruses. Three birds in each group were euthanized by venous bloodletting at 3 days post inoculation (dpi), and the organs or tissues (trachea, lung, liver, spleen, pancreas, kidney, intestine, rectum and bursa of Fabricius) of the birds were collected for virus titration. The organ/tissue samples containing PBS (1 ml of PBS/1 g sample) were first homogenized and then centrifuged at 12,000 rpm/min for five minutes. The supernatant (0.1 ml) was injected into eggs, incubated for 48 h, and the allantoic fluid was harvested to detect the virus in 1% chicken RBCs. Three naive birds were cohoused with three inoculated birds (3:3 donor:contact ratio) within one enclosure at 24 h post inoculation (hpi) to assess virus transmissibility. OP and CL swabs were collected at 1, 3, 5, 7, 9, and 11 dpi for virus titration in the eggs to assess virus shedding. Serum from the inoculated and contact groups was collected at 10, 15, and 21 dpi and tested for haemagglutination inhibition (HI) using 1% chicken RBCs.

### Mouse studies

Six-week-old female BALB/c mice were purchased from Jinan Pengyue Experimental Animal Breeding Co., Ltd. (Shandong, China). Groups of mice were anesthetized with CO_2_ and inoculated intranasally with 10^6^ EID_50_ of the H11 virus or PBS in a volume of 50 μl. The survival and body weights of five mice were monitored daily for 14 days after infection. The nasal turbinates and lungs of three infected mice in each group were harvested at 3 dpi for virus titration in chicken eggs.

## Author contributions

Jinyan Shen and Hong Zhang: data curation, sample collection, methodology, software, validation, visualization | Xiaohong Sun, Mengjing Wang, Mengdi Guan, and Lili Liu: virus isolation, sequencing, animal studies | Yaping Zhang: receptor binding analysis | Yujia Zhai, Wenxi Li, Hongke Xu, Yujiao Xie, Anran Ren, Fengyang Cao, Linghui Kong, and Wenqiang Liu: sample collection, virus isolation, sequencing | Guohua Deng: resources, funding acquisition, writing – review and editing | Jing Guo: funding acquisition, formal analysis, validation, visualization, writing – original draft | Xuyong Li: conceptualization, resources, sample collection, funding acquisition, project Administration, supervision, writing – original draft, review and editing.

## Supplementary Material

Supporting Information.pdf

## References

[1] Kilbourne ED. Taxonomy and comparative virology of the influenza viruses. In: Kilbourne ED, editor. Influenza. Boston, MA: Springer US; 1987. p. 25–32.

[2] Yoon SW, Webby RJ, Webster RG. Evolution and ecology of influenza A viruses. Curr Top Microbiol Immunol. 2014;385:359–375. doi:10.1007/82_2014_396 24990620

[3] Tong S, Li Y, Rivailler P, et al. A distinct lineage of influenza A virus from bats. Proc Natl Acad Sci U S A. 2012;109:4269–4274. doi:10.1073/pnas.1116200109 22371588 PMC3306675

[4] Tong S, Zhu X, Li Y, et al. New world bats harbor diverse influenza A viruses. PLoS Pathog. 2013;9:e1003657. doi:10.1371/journal.ppat.1003657 24130481 PMC3794996

[5] Fereidouni S, Starick E, Karamendin K, et al. Genetic characterization of a new candidate hemagglutinin subtype of influenza A viruses. Emerg Microbes Infect. 2023;12:2225645. doi:10.1080/22221751.2023.2225645 37335000 PMC10308872

[6] Karakus U, Mena I, Kottur J, et al. 2024. H19 influenza A virus exhibits species-specific MHC class II receptor usage. Cell Host Microbe. doi:10.1016/j.chom.2024.05.018 PMC11295516 38889725

[7] Webster RG, Bean WJ, Gorman OT, et al. Evolution and ecology of influenza A viruses. Microbiol Rev. 1992;56:152–179. doi:10.1128/mr.56.1.152-179.1992 1579108 PMC372859

[8] Bi Y, Yang J, Wang L, et al. Ecology and evolution of avian influenza viruses. Curr Biol. 2024;34:R716–R721. doi:10.1016/j.cub.2024.05.053 39106825

[9] Shi J, Zeng X, Cui P, et al. Alarming situation of emerging H5 and H7 avian influenza and effective control strategies. Emerg Microbes Infect. 2023;12:2155072. doi:10.1080/22221751.2022.2155072 36458831 PMC9754034

[10] Gu W, Shi J, Cui P, et al. Novel H5N6 reassortants bearing the clade 2.3.4.4b HA gene of H5N8 virus have been detected in poultry and caused multiple human infections in China. Emerg Microbes Infect. 2022;11:1174–1185. doi:10.1080/22221751.2022.2063076 35380505 PMC9126593

[11] Cui P, Zeng X, Li X, et al. Genetic and biological characteristics of the globally circulating H5N8 avian influenza viruses and the protective efficacy offered by the poultry vaccine currently used in China. Sci China Life Sci. 2022;65:795–808. doi:10.1007/s11427-021-2025-y 34757542

[12] Xie R, Edwards KM, Wille M, et al. The episodic resurgence of highly pathogenic avian influenza H5 virus. Nature. 2023;622:810–817. doi:10.1038/s41586-023-06631-2 37853121

[13] Kwon T, Trujillo JD, Carossino M, et al. Pigs are highly susceptible to but do not transmit mink-derived highly pathogenic avian influenza virus H5N1 clade 2.3.4.4b. Emerg Microbes Infect. 2024;13:2353292. doi:10.1080/22221751.2024.2353292 PMC11132737 38712345

[14] Uyeki TM, Milton S, Abdul Hamid C, et al. 2024. Highly pathogenic avian influenza A(H5N1) virus infection in a dairy farm worker. N Engl J Med. doi:10.1056/NEJMc2405371 38700506

[15] Burrough ER, Magstadt DR, Petersen B, et al. Highly pathogenic avian influenza A(H5N1) clade 2.3.4.4b virus infection in domestic dairy cattle and cats, United States, 2024. Emerg Infect Dis. 2024;30; doi:10.3201/eid3007.240508 PMC11210653 38683888

[16] The Lancet Infectious D. What is the pandemic potential of avian influenza A(H5N1)? Lancet Infect Dis. 2024;24:437. doi:10.1016/s1473-3099(24)00238-x 38670675

[17] Gao R, Cao B, Hu Y, et al. Human infection with a novel avian-origin influenza A (H7N9) virus. N Engl J Med. 2013;368:1888–1897. doi:10.1056/NEJMoa1304459 23577628

[18] Zhang Q, Shi J, Deng G, et al. H7N9 influenza viruses are transmissible in ferrets by respiratory droplet. Science. 2013;341:410–414. doi:10.1126/science.1240532 23868922

[19] Chen H, Yuan H, Gao R, et al. Clinical and epidemiological characteristics of a fatal case of avian influenza A H10N8 virus infection: a descriptive study. Lancet. 2014;383:714–721. doi:10.1016/s0140-6736(14)60111-2 24507376

[20] Huo X, Cui LB, Chen C, et al. Severe human infection with a novel avian-origin influenza A(H7N4) virus. Sci Bull (Beijing). 2018;63:1043–1050. doi:10.1016/j.scib.2018.07.003 32288966 PMC7104738

[21] Zhang Y, Shi J, Cui P, et al. Genetic analysis and biological characterization of H10N3 influenza A viruses isolated in China from 2014 to 2021. J Med Virol. 2023;95:e28476. doi:10.1002/jmv.28476 36609855

[22] Tan X, Yan X, Liu Y, et al. A case of human infection by H3N8 influenza virus. Emerg Microbes Infect. 2022;11:2214–2217. doi:10.1080/22221751.2022.2117097 36000153 PMC9542523

[23] Wang Y, Wang M, Zhang H, et al. Prevalence, evolution, replication and transmission of H3N8 avian influenza viruses isolated from migratory birds in eastern China from 2017 to 2021. Emerg Microbes Infect. 2023;12:2184178. doi:10.1080/22221751.2023.2184178 36913241 PMC10013397

[24] Cui P, Shi J, Yan C, et al. Analysis of avian influenza A (H3N8) viruses in poultry and their zoonotic potential, People’s Republic of China, September 2021 to May 2022. Euro Surveill. 2023;28; doi:10.2807/1560-7917.Es.2023.28.41.2200871 PMC10571489 37824247

[25] Liu K, Ding P, Pei Y, et al. 2022. Emergence of a novel reassortant avian influenza virus (H10N3) in Eastern China with high pathogenicity and respiratory droplet transmissibility to mammals. Sci China Life Sci 65:1024-1035. doi:10.1007/s11427-020-1981-5 34542812

[26] Ge Y, Yao Q, Wang X, et al. Detection of reassortant avian influenza A (H11N9) virus in wild birds in China. Transbound Emerg Dis. 2019;66:1142–1157. doi:10.1111/tbed.13044 30338936

[27] Chaiyawong S, Charoenkul K, Udom K, et al. Genetic characterization of influenza A virus subtypes H11N6, H11N7, and H11N9 isolated from free-grazing ducks, Thailand. Influenza Other Respir Viruses. 2022;16:726–739. doi:10.1111/irv.12960 35001520 PMC9178063

[28] Hurt AC, Vijaykrishna D, Butler J, et al. Detection of evolutionarily distinct avian influenza a viruses in Antarctica. mBio. 2014;5:e01098-14. doi:10.1128/mBio.01098-14 24803521 PMC4010832

[29] Hurt AC, Su YCF, Aban M, et al. Evidence for the introduction, reassortment, and persistence of diverse influenza A viruses in Antarctica. J Virol. 2016;90:9674–9682. doi:10.1128/jvi.01404-16 27535050 PMC5068520

[30] Ogrzewalska M, Couto Motta F, Resende PC, et al. 2022. Influenza A(H11N2) virus detection in fecal samples from Adélie (*Pygoscelis adeliae*) and Chinstrap (*Pygoscelis antarcticus*) Penguins, Penguin Island, Antarctica. Microbiol Spectr 10:e0142722. doi:10.1128/spectrum.01427-22 36121294 PMC9603087

[31] Shinya K, Ebina M, Yamada S, et al. Avian flu: influenza virus receptors in the human airway. Nature. 2006;440:435–436. doi:10.1038/440435a 16554799

[32] de Graaf M, Fouchier RA. 2014. Role of receptor binding specificity in influenza A virus transmission and pathogenesis. Embo J 33:823-841. doi:10.1002/embj.201387442 24668228 PMC4194109

[33] Jiménez-Bluhm P, Karlsson EA, Ciuoderis KA, et al. Avian H11 influenza virus isolated from domestic poultry in a Colombian live animal market. Emerg Microbes Infect. 2016;5:e121. doi:10.1038/emi.2016.121 27924808 PMC5180366

[34] Jiang L, Li J, Cui H, et al. Etiologic characteristics of avian influenza H11 viruses isolated from the live poultry market in southeast coastal region in China. Front Microbiol. 2022;13:1002670. doi:10.3389/fmicb.2022.1002670 36338057 PMC9634483

[35] Kayali G, Barbour E, Dbaibo G, et al. Evidence of infection with H4 and H11 avian influenza viruses among Lebanese chicken growers. PLoS One. 2011;6:e26818. doi:10.1371/journal.pone.0026818 22046370 PMC3203926

[36] Gill JS, Webby R, Gilchrist MJ, et al. Avian influenza among waterfowl hunters and wildlife professionals. Emerg Infect Dis. 2006;12:1284–1286. doi:10.3201/eid1708.060492 16965717 PMC1673214

[37] Hill NJ, Bishop MA, Trovão NS, et al. Ecological divergence of wild birds drives avian influenza spillover and global spread. PLoS Pathog. 2022;18:e1010062. doi:10.1371/journal.ppat.1010062 35588106 PMC9119557

[38] Hicks JT, Edwards K, Qiu X, et al. Host diversity and behavior determine patterns of interspecies transmission and geographic diffusion of avian influenza A subtypes among North American wild reservoir species. PLoS Pathog. 2022;18:e1009973. doi:10.1371/journal.ppat.1009973 35417497 PMC9037922

[39] Wang M, Guo J, Zhang H, et al. Ecological and genetic landscapes of global H12 avian influenza viruses and biological characteristics of an H12N5 virus isolated from wild ducks in Eastern China. Transbound Emerg Dis. 2024;2024:9140418. doi:10.1155/2024/9140418 PMC12017136 40303124

[40] Zhang H, Guo J, Peng P, et al. 2024. Evolution and biological characteristics of the circulated H8N4 avian influenza viruses1. J Integ Agric. doi:10.1016/j.jia.2023.12.033

[41] Wang Y, Zhang H, Wang M, et al. Genetic analysis of a novel H16N3 virus isolated from a migratory gull in China in 2021 and animal studies of infection. Microbiol Spectr. 2022;10:e0248422. doi:10.1128/spectrum.02484-22 36314919 PMC9769943

[42] Wang Y, Wang M, Zhang H, et al. Emergence, evolution, and biological characteristics of H10N4 and H10N8 avian influenza viruses in migratory wild birds detected in Eastern China in 2020. Microbiol Spectr. 2022;10:e0080722. doi:10.1128/spectrum.00807-22 35389243 PMC9045299

[43] Zhao C, Guo J, Zeng X, et al. Novel H7N7 avian influenza viruses detected in migratory wild birds in eastern China between 2018 and 2020. Microbes Infect. 2022;24:105013. doi:10.1016/j.micinf.2022.105013 35580801

[44] Trybus KM, Henry L. Monoclonal antibodies detect and stabilize conformational states of smooth muscle myosin. J Cell Biol. 1989;109:2879–2886. doi:10.1083/jcb.109.6.2879 2480352 PMC2115922

[45] Dugan VG, Chen R, Spiro DJ, et al. 2008. The evolutionary genetics and emergence of avian influenza viruses in wild birds. PLoS Pathog 4:e1000076. doi:10.1371/journal.ppat.1000076 18516303 PMC2387073

[46] Gashaw Adane E, Meseret Bekele A. 2022. One health approach for the control of zoonotic diseases. Zoonoses 2. doi:10.15212/ZOONOSES-2022-0037

[47] Guha D, Ruiyun L, Emmanuel C, et al. 2022. The animal origin of major human infectious diseases: What can past epidemics teach us about preventing the next pandemic? Zoonoses 2. doi:10.15212/ZOONOSES-2021-0028

[48] Bui C, Bethmont A, Chughtai AA, et al. A systematic review of the comparative epidemiology of avian and human influenza A H5N1 and H7N9 – lessons and unanswered questions. Transbound Emerg Dis. 2016;63:602–620. doi:10.1111/tbed.12327 25644240

[49] Yuen KY. Another avian influenza A subtype jumping into human: this time is H7N4. Sci Bull (Beijing). 2018;63:1025–1026. doi:10.1016/j.scib.2018.08.002 36755450

[50] Liu K, Qi X, Bao C, et al. 2024. Novel H10N3 avian influenza viruses: a potential threat to public health. Lancet Microbe. doi:10.1016/s2666-5247(23)00409-3 38309285

[51] Sun H, Li H, Tong Q, et al. 2023. Airborne transmission of human-isolated avian H3N8 influenza virus between ferrets. Cell 186:4074-4084.e11. doi:10.1016/j.cell.2023.08.011 37669665

[52] Zhu W, Chen Q, Xu X, et al. Biological features of human influenza A(H3N8) viruses in China. J Med Virol. 2023;95:e28912. doi:10.1002/jmv.28912 37403888

[53] Shi J, Deng G, Liu P, et al. Isolation and characterization of H7N9 viruses from live poultry markets – implication of the source of current H7N9 infection in humans. Chin Sci Bull. 2013;58:1857–1863. doi:10.1007/s11434-013-5873-4

[54] Yang R, Sun H, Gao F, et al. Human infection of avian influenza A H3N8 virus and the viral origins: a descriptive study. Lancet Microbe. 2022;3:e824–e834. doi:10.1016/s2666-5247(22)00192-6 36115379

[55] Gao R, Zheng H, Liu K, et al. 2021. Genesis, evolution and host species distribution of influenza A (H10N3) virus in China. J Infect 83:607-635. doi:10.1016/j.jinf.2021.08.021 34416256

[56] Fusaro A, Zecchin B, Giussani E, et al. 2024. High pathogenic avian influenza A(H5) viruses of clade 2.3.4.4b in Europe-Why trends of virus evolution are more difficult to predict. Virus Evol 10:veae027. doi:10.1093/ve/veae027 38699215 PMC11065109

[57] Hood G, Roche X, Brioudes A, et al. A literature review of the use of environmental sampling in the surveillance of avian influenza viruses. Transbound Emerg Dis. 2021;68:110–126. doi:10.1111/tbed.13633 32652790 PMC8048529

[58] Tomás G, Marandino A, Panzera Y, et al. Highly pathogenic avian influenza H5N1 virus infections in pinnipeds and seabirds in Uruguay: implications for bird-mammal transmission in South America. Virus Evol. 2024;10:veae031. doi:10.1093/ve/veae031 38756986 PMC11096771

[59] Meade PS, Bandawane P, Bushfield K, et al. Detection of clade 2.3.4.4b highly pathogenic H5N1 influenza virus in New York City. J Virol. 2024;98:e0062624. doi:10.1128/jvi.00626-24 38747601 PMC11237497

[60] Li X, Shi J, Guo J, et al. Genetics, receptor binding property, and transmissibility in mammals of naturally isolated H9N2 avian influenza viruses. PLoS Pathog. 2014;10:e1004508. doi:10.1371/journal.ppat.1004508 25411973 PMC4239090

[61] Song J, Feng H, Xu J, et al. The PA protein directly contributes to the virulence of H5N1 avian influenza viruses in domestic ducks. J Virol. 2011;85:2180–2188. doi:10.1128/jvi.01975-10 21177821 PMC3067757

[62] Sun H, Pu J, Hu J, et al. Characterization of clade 2.3.4.4 highly pathogenic H5 avian influenza viruses in ducks and chickens. Vet Microbiol. 2016;182:116–122. doi:10.1016/j.vetmic.2015.11.001 26711037

[63] Guo J, Wang Y, Zhao C, et al. 2021. Molecular characterization, receptor binding property, and replication in chickens and mice of H9N2 avian influenza viruses isolated from chickens, peafowls, and wild birds in eastern China. Emerg Microbes Infect 10:2098-2112. doi:10.1080/22221751.2021.1999778 34709136 PMC8592596

[64] Liu LL, Fang B, Yu X, et al. Strengthened monitoring of H5 avian influenza viruses in external environment in Hubei, 2018. Curr Med Sci. 2020;40:63–68. doi:10.1007/s11596-020-2147-7 32166666

[65] Jonges M, Welkers MR, Jeeninga RE, et al. Emergence of the virulence-associated PB2 E627 K substitution in a fatal human case of highly pathogenic avian influenza virus A(H7N7) infection as determined by Illumina ultra-deep sequencing. J Virol. 2014;88:1694–1702. doi:10.1128/jvi.02044-13 24257603 PMC3911586

[66] Xie J, Chen Y, Cai G, et al. 2023. Tree visualization by one table (tvBOT): a web application for visualizing, modifying and annotating phylogenetic trees. Nucl Acids Res 51:W587–wW592. doi:10.1093/nar/gkad359 37144476 PMC10320113

