# Supplementary material for: Evolution and biological characteristics of H11 avian influenza viruses isolated from migratory birds and pigeons
Source: Emerg Microbes Infect. 2024 Sep 9;13(1):2398641. doi: 10.1080/22221751.2024.2398641 (PMC11622381; doi:10.1080/22221751.2024.2398641)
Supplement: Supporting Information.pdf [file TEMI_A_2398641_SM0843.pdf]

Supplemental Material

Supplemental figures legends

Figure S1. Host reservoirs of each subtype combination of the global H11 viruses. The host species of each subtype of the H11 viruses (H11N1, H11N2, H11N3, H11N4, H11N5, H11N6, H11N7, H11N8, H11N9) were categorized according to the isolation information of the available sequences in GISAID and GenBank databases.

Figure S2. Locations of the sampling sites in eastern China. Four sampling sites were involved in this study, including one live poultry market and three wild birds habitats.

Figure S3. Phylogenetic trees of the NA (N2 and N3) genes of the H11N2 and H11N3 viruses.

Figure S4. Phylogenetic trees of the internal genes (PB2, PB1, PA, NP, M, and NS) of the H11 viruses.

Figure S5. HI antibodies in the serum of the inoculated and contact birds. Serum samples from inoculated and contact ducks (A-C) and chickens (D-F) were collected at 10, 15, and 21 dpi to detect HI antibodies. The dashed lines indicate the lower limit of HI antibody detection.

Table S1. List of species of birds and mammals mentioned in this study. Bird names taxonomy follows the Clements Checklist.

Order	Family	Genus	Scientific name	English name
Artiodactyla	*Suidae*	*Sus*	*Sus scrofa*	Swine
Anseriformes	*Anatidae*			Waterfowl*
				Teal*
		Aix	*Aix galericulata*	Mandarin duck
			Aix sponsa	Wood duck
		Anas		Shoveler*
			Anas acuta	Northern pintail
			Anas crecca	Green-winged teal
			Anas erythrorhyncha	Red-billed duck
			Anas flavirostris	Yellow-billed teal
			Anas georgica	Yellow-billed pintail
			Anas gracilis	Grey teal
			Anas platyrhynchos	Mallard
			Anas platyrhynchos (Domestic type)	Duck
			Anas platyrhynchos x rubripes	Mallard x black duck
			Anas poecilorhyncha	Indian spot-billed duck
			Anas rubripes	American black duck
			Anas sparsa	African black duck
			Anas superciliosa	Pacific black duck
		Anser	*Anser albifrons*	White-fronted goose
			Anser anser (Domestic type)	Goose
			Anser brachyrhynchus	Pink-footed goose
			Anser canagicus	Emperor goose
			Anser fabalis	Taiga bean goose
			Anser rossii	Ross's goose
		Aythya	*Aythya affinis*	Lesser scaup
			Aythya americana	Redhead duck
			Aythya collaris	Ring-necked duck
			Aythya ferina	Common pochard
			Aythya marila	Greater scaup
		Branta	*Branta bernicla*	Brant
		Bucephala	*Bucephala albeola*	Bufflehead
			Bucephala clangula	Common goldeneye
		Cairina	*Cairina moschata*	Muscovy duck
		Calidris	*Calidris acuminata*	Sharp-tailed sandpiper
			Calidris alba	Sanderling
			Calidris alpina	Dunlin
			Calidris canutus	Red knot
			Calidris minutilla	Least sandpiper
			Calidris pusilla	Semipalmated sandpiper

		Chenonetta	*Chenonetta jubata*	Australian wood duck
		Cygnus	*Cygnus cygnus*	Whooper swan
		Heteronetta	*Heteronetta atricapilla*	Black-headed duck
		Mareca	*Mareca americana*	Widgeon
			Mareca falcata	Falcated duck
			Mareca penelope	Eurasian wigeon
			Mareca strepera	Gadwall
		Polysticta	*Polysticta stelleri*	Steller's eider
		Sibirionetta	*Sibirionetta formosa*	Baikal teal
		Spatula	*Spatula clypeata*	Northern shoveler
			Spatula cyanoptera	Cinnamon teal
			Spatula discors	Blue-winged teal
			Spatula platalea	Red shoveler
	Ardeidae	*Egretta*		Egret*
Charadriiformes				Shorebird*
	Alcidae	*Uria*	*Uria aalge*	Common murre
			Uria lomvia	Thick-billed murre
	Laridae	*Chroicocephalus*	*Chroicocephalus ridibundus*	Black-headed gull
		Larus		Gull*
			Larus argentatus	Herring gull
			Larus fuscus	Lesser black-backed gull
			Larus glaucescens	Glaucous-winged gull
			Larus glaucoides	Iceland gull
		Leucophaeus	*Leucophaeus atricilla*	Laughing gull
		Sterna	*Sterna hirundo*	Common tern
	Recurvirostridae	*Himantopus*	*Himantopus mexicanus*	Black-necked stilt
	Scolopacidae	*Arenaria*	*Arenaria interpres*	Ruddy turnstone
Ciconiiformes	*Ciconiidae*	*Ciconia*	*Ciconia boyciana*	Oriental white stork
			Ciconia ciconia	White stork
Columbiformes	*Columbidae*	*Columba*	*Columba livia*	Pigeon
Galliformes	*Numididae*			Guinea fowl*
		Numida	*Numida meleagris*	Helmeted guineafowl
	Phasianidae	*Gallus*	*Gallus gallus*	Chicken
		Meleagris	*Meleagris gallopavo*	Turkey
Gruiformes	*Gruidae*			Cranes*
	Rallidae	*Fulica*	*Fulica atra*	Eurasian coot
Sphenisciformes	*Spheniscidae*	*Pygoscelis*	*Pygoscelis adeliae*	Adelie penguin
			Pygoscelis antarcticus	Chinstrap penguin
			Pygoscelis papua	Gentoo penguin
Struthioniformes	*Struthionidae*	*Struthio*	*Struthio camelus*	Ostrich

*Genus or identification at broad level.

Waterfowl: Family *Anatidae*; Teal: Family *Anatidae*; Shoveler: Genus *Anas*; Egret: Genus *Egretta*; Shorebird: Order *Charadriiformes*; Gull: Genus *Larus*; Guinea fowl: Family *Numididae*; Cranes: Family *Gruidae*.

Table S2 Information on all the H11 viruses available in GISAID and GenBank that have been detected in China since 2002.

NO.	Isolate Name	Subtype	Location	Host	Isolate Id/Accession	Collection Date
1	A/duck/Yangzhou/906/2002	H11N2	Yangzhou	duck	EPI_ISL_8517	2002
2	A/spotbill duck/Xuyi/6/2005	H11N2	Xuyi	spotbill duck	EPI_ISL_11854	2005
3	A/Baikal Teal/Hongze/14/2005	H11N9	Hongze	Baikal Teal	EPI_ISL_11856	2005
4	A/wild waterfowl/Hong Kong/MPC729/2006	H11N9	Hong Kong	wild waterfowl	EPI_ISL_146348	2006
5	A/mallard/Sanjiang/148/2006	H11N9	Sanjiang	mallard	EPI_ISL_378701	2006-10-22
6	A/Oriental white stork/Zhalong/183/2006	H11N9	Zhalong	Oriental white stork	EPI_ISL_379001	2006-10-22
7	A/duck/Yunnan/1282/2007	H11N9	Yunnan	duck	EPI_ISL_146332	2007
8	A/duck/Guangdong/4323/2007	H11N9	Guangdong	duck	EPI_ISL_146326	2007
9	A/northern pintail/Hong Kong/MPC2085/2007	H11N9	Hong Kong	northern pintail	EPI_ISL_146350	2007
10	A/common teal/Hong Kong/MPD322/2007	H11N9	Hong Kong	common teal	EPI_ISL_146349	2007
11	A/duck/Yunnan/2908/2009	H11N9	Yunnan	duck	EPI_ISL_146333	2009
12	A/duck/Jiangxi/11278/2009	H11N9	Jiangxi	duck	EPI_ISL_146319	2009
13	A/northern pintail/Hong Kong/MPJ851/2009	H11N9	Hong Kong	northern pintail	EPI_ISL_146344	2009
14	A/northern shoveler/Hong Kong/MPK276/2009	H11N9	Hong Kong	northern shoveler	EPI_ISL_146351	2009
15	A/eurasian wigeon/Hong Kong/MPK653/2009	H11N9	Hong Kong	eurasian wigeon	EPI_ISL_146353	2009
16	A/eurasian wigeon/Hong Kong/MPK655/2009	H11N9	Hong Kong	eurasian wigeon	EPI_ISL_146345	2009
17	A/chicken/Nanjing/908/2009	H11N2	Nanjing	chicken	EPI_ISL_130432	2009-03-01
18	A/duck/Jiangxi/k0701/2009	H11N2	Jiangxi	duck	EPI_ISL_94966	2009-07
19	A/environment/Jiangxi/28/2009	H11N9	Jiangxi	environment	EPI_ISL_140708	2009-07-08
20	A/environment/Jiangxi/26/2009	H11N9	Jiangxi	environment	EPI_ISL_140707	2009-07-08
21	A/wild waterfowl/Hong Kong/MPJ1815/2010	H11N9	Hong Kong	wild waterfowl	EPI_ISL_146362	2010
22	A/wild waterfowl/Hong Kong/MPL1060/2011	H11N9	Hong Kong	wild waterfowl	EPI_ISL_146360	2011
23	A/duck/Guizhou/1078/2011	H11N9	Guizhou	duck	EPI_ISL_146402	2011
24	A/duck/Jiangxi/21714/2011	H11N9	Jiangxi	duck	EPI_ISL_146320	2011
25	A/common teal/Hong Kong/MPL1075/2011	H11N9	Hong Kong	common teal	EPI_ISL_146368	2011
26	A/duck/Jiangsu/10-d4/2011	H11N3	Jiangsu	duck	EPI_ISL_127581	2011-10
27	A/duck/Hunan/S4013/2011	H11N9	Hunan	duck	EPI_ISL_143940	2011-11-09
28	A/duck/Hunan/S4137/2011	H11N9	Hunan	duck	EPI_ISL_143946	2011-11-11
29	A/duck/Hunan/S4474/2011	H11N9	Hunan	duck	EPI_ISL_143951	2011-11-13
30	A/duck/Hunan/S4443/2011	H11N9	Hunan	duck	EPI_ISL_143950	2011-11-13
31	A/duck/Jiangxi/22620/2012	H11N9	Jiangxi	duck	EPI_ISL_146325	2012
32	A/duck/Jiangxi/22597/2012	H11N9	Jiangxi	duck	EPI_ISL_146324	2012
33	A/duck/Jiangxi/22537/2012	H11N9	Jiangxi	duck	EPI_ISL_146323	2012
34	A/environment/Hunan/S1798/2012	H11N2	Hunan	environment	EPI_ISL_143953	2012-03-24
35	A/duck/Hunan/S1607/2012	H11N9	Hunan	duck	EPI_ISL_143937	2012-03-24
36	A/duck/Zhejiang/71750/2013	H11N9	Zhejiang	duck	KR864826-KR864833	2013-07
37	A/duck/Zhejiang/727D2/2013	H11N3	Zhejiang	duck	EPI_ISL_221397	2013-07-27
38	A/duck/Zhejiang/727D26/2013	H11N3	Zhejiang	duck	EPI_ISL_221396	2013-07-27
39	A/duck/Zhejiang/727D25/2013	H11N3	Zhejiang	duck	EPI_ISL_221395	2013-07-27
40	A/duck/Zhejiang/727D22/2013	H11N3	Zhejiang	duck	EPI_ISL_221394	2013-07-27
41	A/duck/Zhejiang/727D9/2013	H11N3	Zhejiang	duck	EPI_ISL_221393	2013-07-27
42	A/duck/Zhejiang/727D7/2013	H11N3	Zhejiang	duck	EPI_ISL_221392	2013-07-27
43	A/chicken/Wuxi/JYJN132/2014	H11N2	Wuxi	chicken	EPI_ISL_277044	2013-12-24
44	A/duck/Wuxi/JYJN126/2014	H11N2	Wuxi	duck	EPI_ISL_277043	2013-12-24
45	A/duck/Wuxi/JYJN203/2014	H11N2	Wuxi	duck	EPI_ISL_277045	2014-02-15
46	A/duck/Jiangsu/J1435/2014	H11N2	Jiangsu	duck	EPI_ISL_209504	2014-04-28
47	A/wild birds/Hubei/89/2014	H11N9	Hubei	wild birds	EPI_ISL_505081	2014-12-08
48	A/Anser fabalis/China/664/2014	H11N8	China	Anser fabalis	EPI_ISL_368348	2014-12-19
49	A/wild bird/Anhui Shengjin Lake/S119/2014	H11N9	Anhui Shengjin Lake	wild bird	EPI_ISL_378703	2014-12-24
50	A/wild bird/Anhui Caizi Lake/L306/2014	H11N9	Anhui Caizi Lake	wild bird	EPI_ISL_378702	2014-12-24
51	A/wild bird/Wuhan/CDHN173/2015	H11N9	Wuhan	wild bird	EPI_ISL_205149	2015-01
52	A/wild bird/Wuhan/CDHN22/2015	H11N9	Wuhan	wild bird	EPI_ISL_205148	2015-01
53	A/wild bird/Wuhan/CDHN01/2015	H11N9	Wuhan	wild bird	EPI_ISL_205147	2015-01
54	A/Bean Goose/Hubei/chenhu V108/2015	H11N9	Hubei	Bean Goose	EPI_ISL_212414	2015-01-09
55	A/Anas falcata/China/D257/2015	H11N8	China	Anas falcata	EPI_ISL_368347	2015-02-06
56	A/Bean Goose/Hubei/chenhu VIII49/2015	H11N8	Hubei	Bean Goose	EPI_ISL_212427	2015-03-09
57	A/Bean Goose/Hubei/chenhu VIII10/2015	H11N8	Hubei	Bean Goose	EPI_ISL_212426	2015-03-09
58	A/duck/Hunan/04.14 YYGK440-P/2015	H11N2	Hunan	duck	EPI_ISL_199117	2015-04-14
59	A/Bean Goose/Hubei/chenhu XVI4-2/2016	H11N9	Hubei	Bean Goose	EPI_ISL_217484	2016-01-27
60	A/Bean Goose/Hubei/chenhu XVI4-1/2016	H11N9	Hubei	Bean Goose	EPI_ISL_217483	2016-01-27
61	A/Bean Goose/Hubei/chenhu XVI270-1/2016	H11N5	Hubei	Bean Goose	EPI_ISL_217877	2016-02-26
62	A/bean goose/Hubei/SZY200/2016	H11N9	Hubei	bean goose	KX121185-KX121192	2016-02-26
63	A/Eurasian coot/Shanghai/PD112440/2016	H11N9	Shanghai	Eurasian coot	MN049550-MN049557	2016-11-24
64	A/environment/Fujian/S1XA33/2017	H11N3	Fujian	environment	EPI_ISL_285340	2017-03
65	A/duck/Fujian/SD061/2017	H11N3	Fujian	duck	EPI_ISL_285338	2017-03
66	A/Eurasion wigeon/Shanghai/NH101834/2017	H11N2	Shanghai	Eurasion wigeon	EPI_ISL_499087	2017-10-18
67	A/environment/Fujian/EV01/2020	H11N3	Fujian	environment	EPI_ISL_14874795	2020-01-14

The information of these H11 viruses were available from the the databases, but did not include the viruses detected in this study.

Table S3. Information on the twenty H11 viruses included in this study.

NO.	Full name	Abbreviation	Subtype	Location	Date	Host	Specimen source	Sample numbers
1	A/wild bird/Shandong/W2295/2018	WB/W2295/18	H11N2	a	31 Oct 2018	Wild bird	Fecal droppings	811
2	A/wild bird/Shandong/W2048/2018	WB/W2048/18	H11N9					
3	A/wild bird/ShandongW/2309/2018	WB/W2309/18	H11N2					
4	A/wild bird/Shandong/W2508/2018	WB/W2508/18	H11N9					
5	A/wild bird/Shandong/W2241/2018	WB/W2241/18	H11N9					
6	A/pigeon/Shandong/D173/2019	PG/D173/19	H11N2	c	11 Jan 2019	Pigeon	Oropharyngeal and cloacal swabs	41
7	A/swan/Shandong/W756/2019	SW/W756/19	H11N9	b	13 Jan 2019	Swan	Fecal droppings	420
8	A/wild bird/Shandong/W2282/2019	WB/W2282/19	H11N9	a	1 Mar 2019	Wild bird	Fecal droppings	968
9	A/egret/Shandong/W12809/2019	ER/W12809/19	H11N2	a	25 Dec 2019	Egret<br>Wild duck	Fecal droppings	1132
10	A/wild duck/Shandong/W12782/2019	WD/W12782/19	H11N2					
11	A/wild duck /Shandong/W12596/2019	WD/W12596/19	H11N9					
12	A/wild duck/Shandong/W12626/2019	WD/W12626/19	H11N9					
13	A/eurasian coot/Shandong/W6086/2020	EC/W6086/20	H11N9	a	31 Dec 2020	Eurasian coot	Fecal droppings	987
14	A/swan/Shandong/W3701/2021	SW/W3701/21	H11N2	b	23 Dec 2021	Whooper swan	Fecal droppings	726
15	A/swan/Shandong/W4154/2021	SW/W4154/21	H11N3					
16	A/swan/Shandong/W4153/2021	SW/W4153/21	H11N3					
17	A/gull/Shandong/W1806/2022	GL/W1806/22	H11N9	b	19 Feb 2022	Gull	Fecal droppings	804
18	A/gull/Shandong/W1805/2022	GL/W1805/22	H11N9					
19	A/gull/Shandong/W1807/2022	GL/W1807/22	H11N9					
20	A/egret/Shandong/W2747/2022	ER/W2747/22	H11N9	d	20 Aug 2022	Egret	Fecal droppings	560

a: Yellow River Delta wetland, Shandong, China b: Swan Lake wetland, Shandong, China c: Live poultry market, Shandong, China d: Tuhai River wetland, Shandong, China

Table S4. Isolate IDs and accession numbers of the twenty H11 viruses in this study.

Full name	Isolate ID	accession
A/wild bird/Shandong/W2295/2018	EPI_ISL_19057187	EPI3217124-EPI3217131
A/wild bird/Shandong/W2048/2018	EPI_ISL_19057188	EPI3217132-EPI3217139
A/wild bird/ShandongW/2309/2018	EPI_ISL_19057189	EPI3217351-EPI3217358
A/wild bird/Shandong/W2508/2018	EPI_ISL_19057421	EPI3217411、EPI3217415、EPI3217444、EPI3217473、EPI3217497、EPI3217550、EPI3217575、EPI3217582
A/wild bird/Shandong/W2241/2018	EPI_ISL_19057496	EPI3217583-EPI3217590
A/pigeon/Shandong/D173/2019	EPI_ISL_19059350	EPI3220353-EPI3220359、EPI3221712
A/swan/Shandong/W756/2019	EPI_ISL_19057497	EPI3217591-EPI3217598
A/wild bird/Shandong/W2282/2019	EPI_ISL_19057498	EPI3217599-EPI3217606
A/egret/Shandong/W12809/2019	EPI_ISL_19057531	EPI3217607-EPI3217614
A/wild duck/Shandong/W12782/2019	EPI_ISL_19058876	EPI3219533-EPI3219540
A/wild duck /Shandong/W12596/2019	EPI_ISL_19058877	EPI3219541-EPI3219548
A/wild duck/Shandong/W12626/2019	EPI_ISL_19058887	EPI3219555-EPI3219563
A/eurasian coot/Shandong/W6086/2020	EPI_ISL_19059060	EPI3219568-EPI3219575
A/swan/Shandong/W3701/2021	EPI_ISL_19057094	EPI3217094-EPI3217101
A/swan/Shandong/W4154/2021	EPI_ISL_19057186	EPI3217116-EPI3217123
A/swan/Shandong/W4153/2021	EPI_ISL_19057153	EPI3217102-EPI3217109
A/gull/Shandong/W1806/2022	EPI_ISL_19059062	EPI3219576-EPI3219583
A/gull/Shandong/W1805/2022	EPI_ISL_19059063	EPI3219584-EPI3219591
A/gull/Shandong/W1807/2022	EPI_ISL_19059064	EPI3219592-EPI3219597
A/egret/Shandong/W2747/2022	EPI_ISL_19059065	EPI3219600-EPI3219607

Table S5. Molecular characteristics of the H11 viruses in this study.

	**HA (H3 Numbering)**								PB2		PB1		PA	NP	M1		NS1
	**Cleavage site**	155	160	193	222	226	227	228	627	701	207	436	515	41	30	215	149
WB/W2295/18	PAIASR/GLF	I	T	D	K	Q	A	G	E	D	K	Y	T	I	D	A	A
WB/W2048/18	PAIASR/GLF	I	T	D	K	Q	A	G	E	D	K	Y	T	I	D	A	A
WB/W2309/18	PAIASR/GLF	I	T	D	K	Q	A	G	E	D	K	Y	T	I	D	A	A
WB/W2508/18	PAIASR/GLF	I	T	D	K	Q	A	G	E	D	K	Y	T	I	D	A	A
WB/W2241/18	PAIASR/GLF	I	T	D	K	Q	A	G	E	D	K	Y	T	I	D	A	A
PG/D173/19	PAIASR/GLF	I	T	D	K	Q	A	G	V	D	K	/	T	I	D	A	A
SW/W756/19	PAIASR/GLF	I	T	D	K	Q	A	G	E	D	K	Y	T	I	D	A	A
WB/W2282/19	PAIASR/GLF	I	T	D	K	Q	A	G	E	D	K	Y	T	I	D	A	A
ER/W12809/19	PAIASR/GLF	I	T	D	K	Q	A	G	E	D	K	Y	T	I	D	A	A
WD/W12782/19	PAIASR/GLF	I	T	D	K	Q	A	G	E	D	K	Y	T	I	D	A	A
WD/W12596/19	PAIASR/GLF	I	T	D	K	Q	A	G	E	D	R	Y	T	I	D	A	A
WD/W12626/19	PAIASR/GLF	I	T	D	K	Q	A	G	E	D	K	Y	T	I	D	A	A
EC/W6086/20	PAIASR/GLF	I	T	D	K	Q	A	G	E	D	K	Y	T	I	D	A	A
SW/W3701/21	PAIASR/GLF	I	T	D	K	Q	A	G	E	D	K	Y	T	I	D	A	A
SW/W4154/21	PAIASR/GLF	I	T	N	E	Q	A	G	E	D	K	Y	T	I	D	A	A
SW/W4153/21	PAIASR/GLF	I	T	N	K	Q	A	G	E	D	K	Y	T	I	D	A	A
GL/W1806/22	PSIASR/GLF	I	T	N	K	Q	A	G	E	D	K	Y	T	I	D	A	A
GL/W1805/22	PSIASR/GLF	I	T	N	K	Q	A	G	E	D	K	Y	T	I	D	A	A
GL/W1807/22	PSIASR/GLF	I	T	N	K	Q	A	G	E	D	K	Y	T	I	D	A	A
ER/W2747/22	PSIASR/GLF	I	T	N	K	Q	A	G	E	D	K	Y	T	I	D	A	A

Table S6. Gene segment-specific primers used in this study.

Primer name	Primer sequence（5’-3’）
H11HA-F	AACAATGTCACRGTAACT
H11HA-R	GGCGATTGATTTTAGATT
NA (N2)-F	AAGCAGGAGTGAAAATGAATCCG
NA (N2)-R	ATGTTCGCTCCATCAGGCCA
NA (N3)-F	TGCGAGATGAATCCAAATCA
NA (N3)-R	TTGGGCATAAACCCAATGTT
NA (N9)-F	TCTATGCACTTCAGCCAC
NA (N9)-R	ACTGGAACACATCGMTAC
PA1-F	AGCRAAAGCAGGTACTGATYCAAAA
PA1-R	ATCCARCTYGARTCWGTCAATTC
PA2-F	GGCACCRGARAAAGTRGACTTTGA
PA2-R	AGTAGAAACAAGGTACTTTTTTGGA
PB11-F	AGCRAAAGCAGGCAAACCATTTGAA
PB11-R	GAGGATTGGAGYCCRTCCCACCA
PB12-F	ATGATGATGGGCATGTTCAACATG
PB12-R	AGTAGAAACAAGGCATTTTTTCA
PB21-F	AGCRAAAGCAGGTCAAWTATATTCA
PB21-R	ACTGCYTTTATCATGCAMTCYTC
PB22-F	GCAACRGCTATYYTRAGGAAAGC
PB22-R	AGTAGAAACAAGGTCGTTTTTAAA
M-F	AGCRAAAGCAGGTAGGTRTTKAAA
M-R	ATGAGAAACAAGGTAGTTTTTTA
NP-F	AGCRAAAGCAGGGTAGATAATCA
NP-R	AGTAGAAACAAGGGTATTTTTCTT
NS-F	AGCAAAAGCAGGGTGACAAA
NS-R	AGAAACAAGGGTGTTTTTTA

Figure S1

H11N1

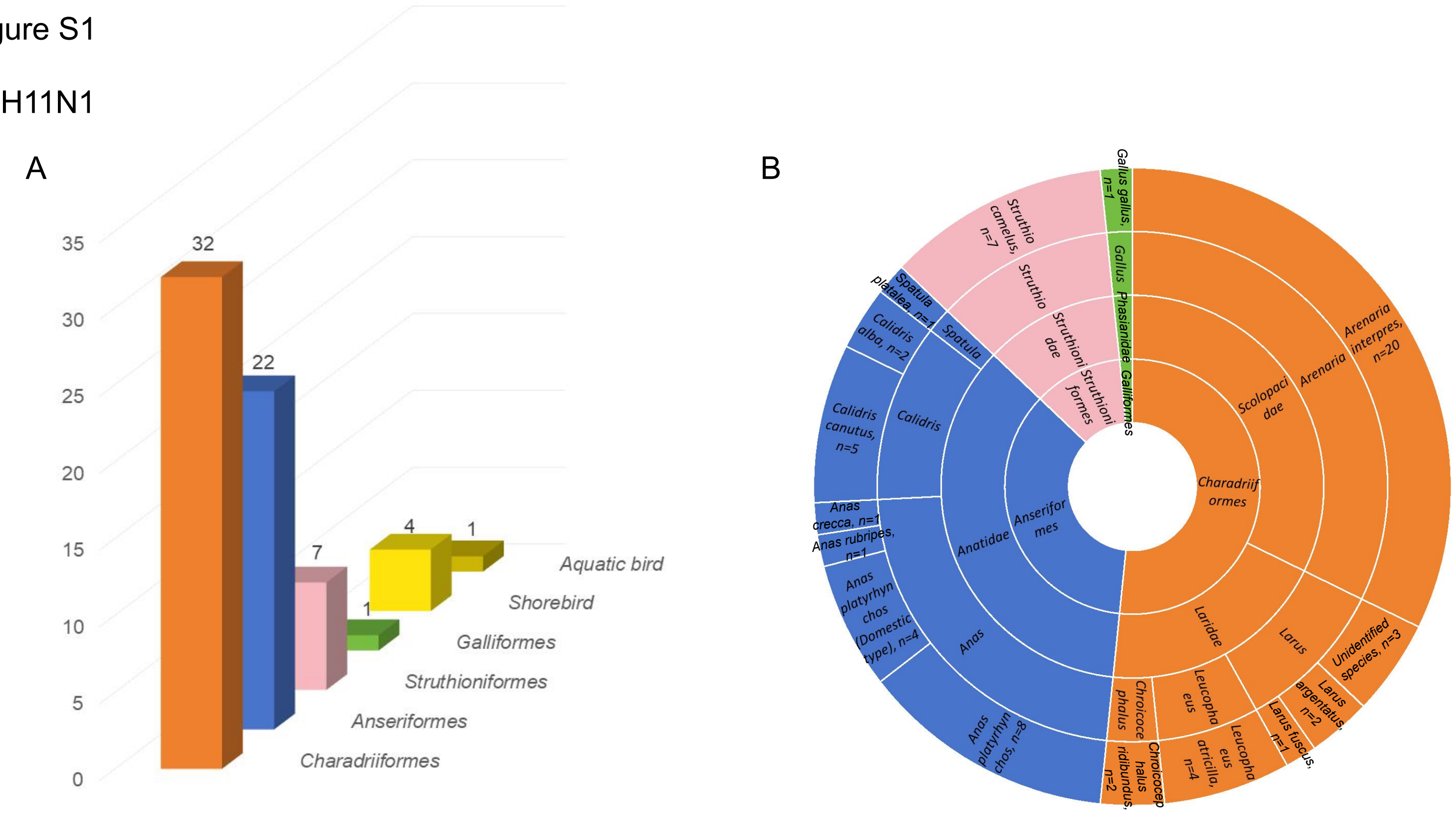

H11N2

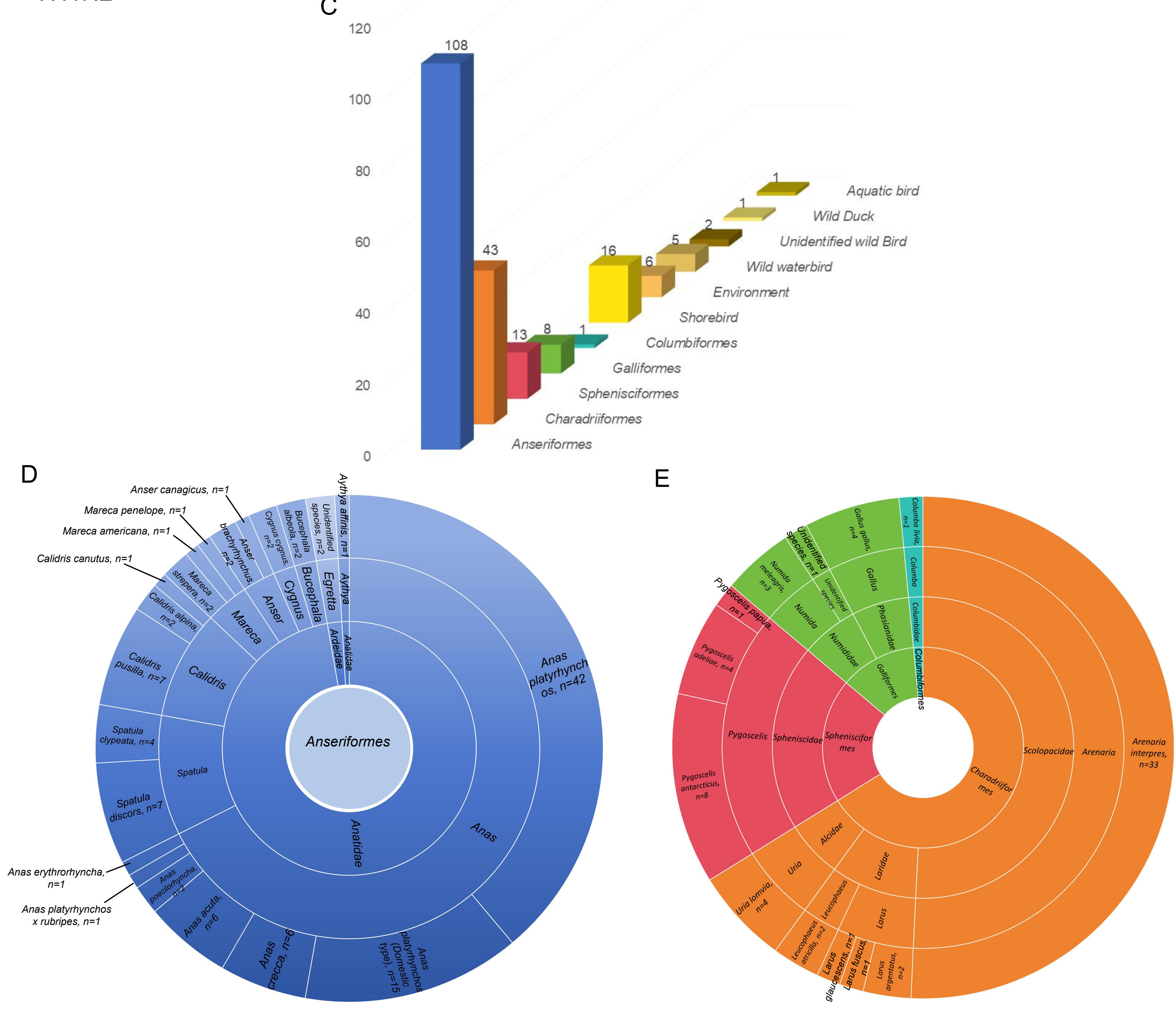

H11N3

F

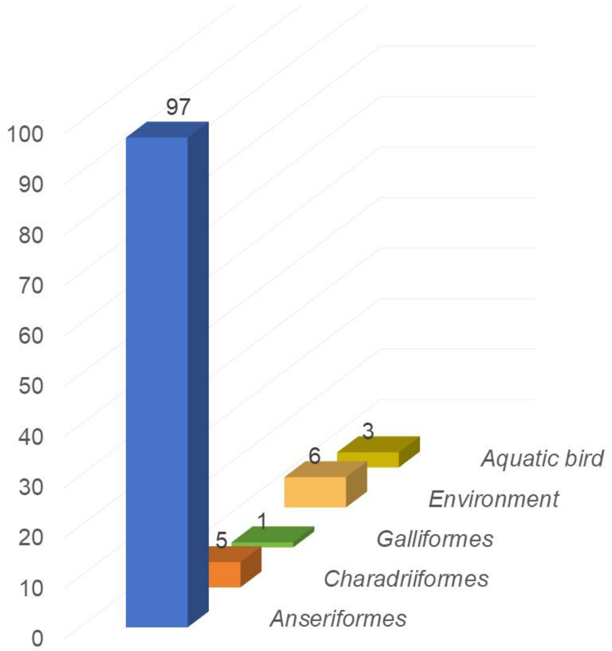

G

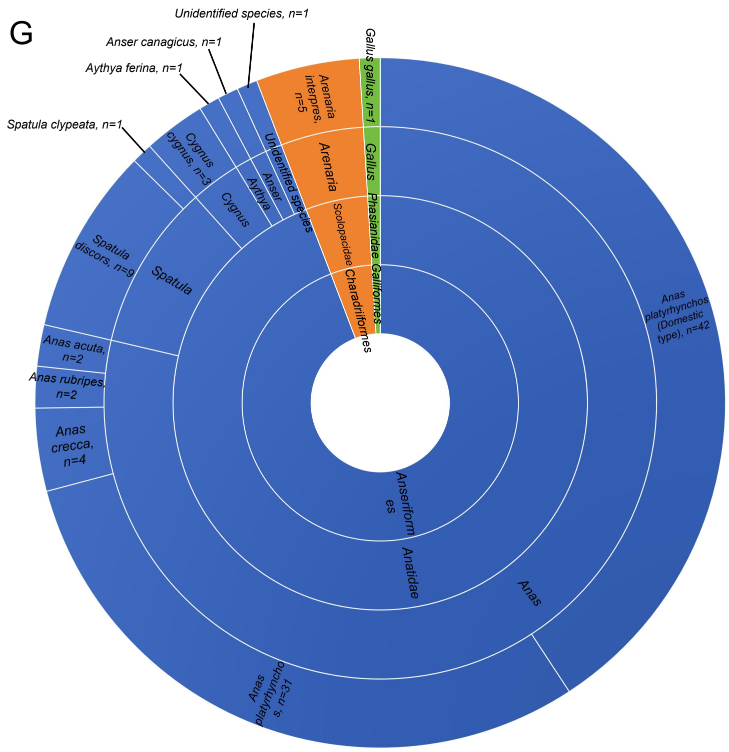

H11N4-H11N8

H

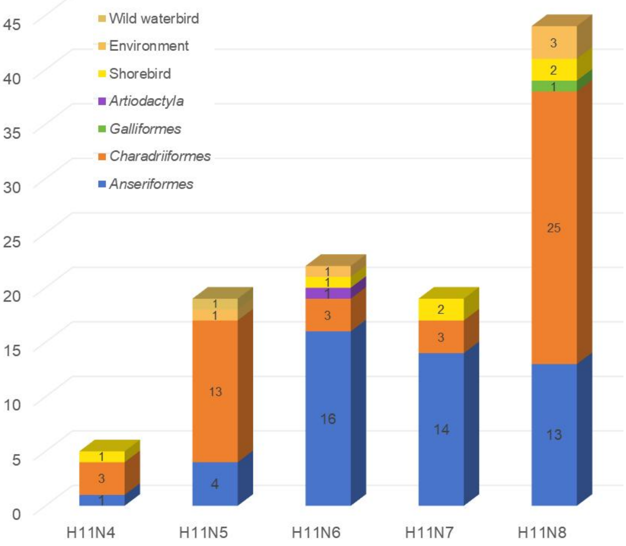

I

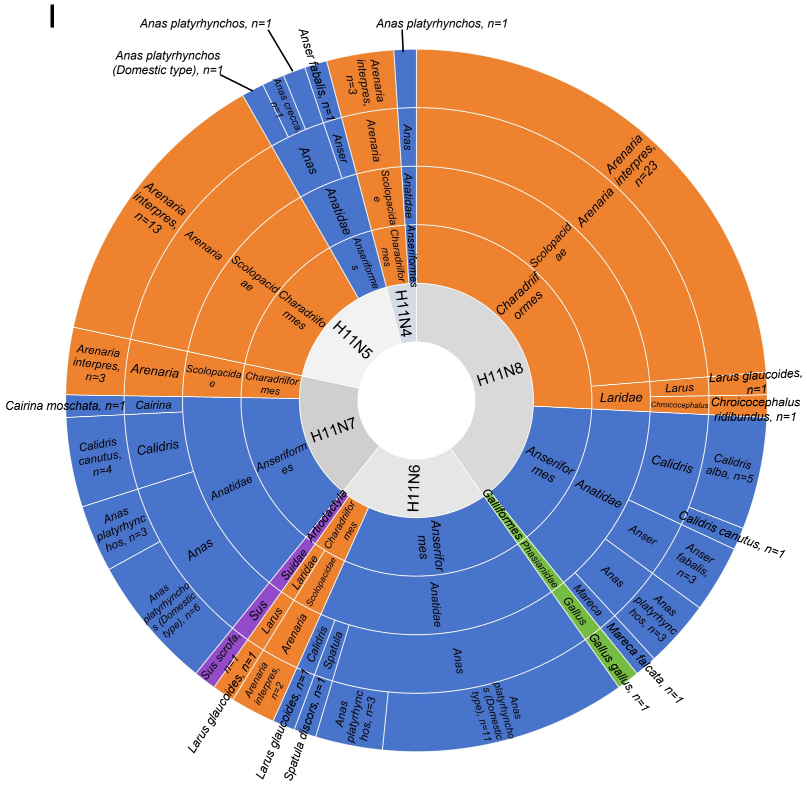

H11N9

J

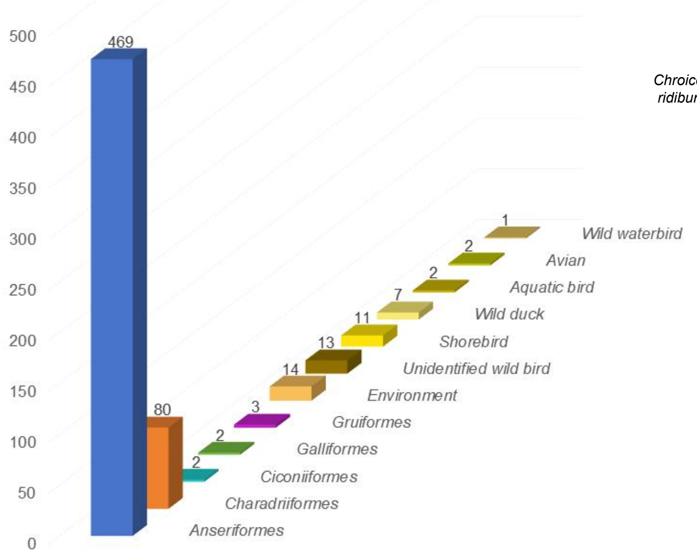

500
450
400
350
300
250
200
150
100
50
0
469
80
2
2
3
14
13
11
7
2
2
1
Wild waterbird
Avian
Aquatic bird
Wild duck
Shorebird
Unidentified wild bird
Environment
Gruiformes
Galliformes
Ciconiiformes
Charadriiformes
Anseriformes

K

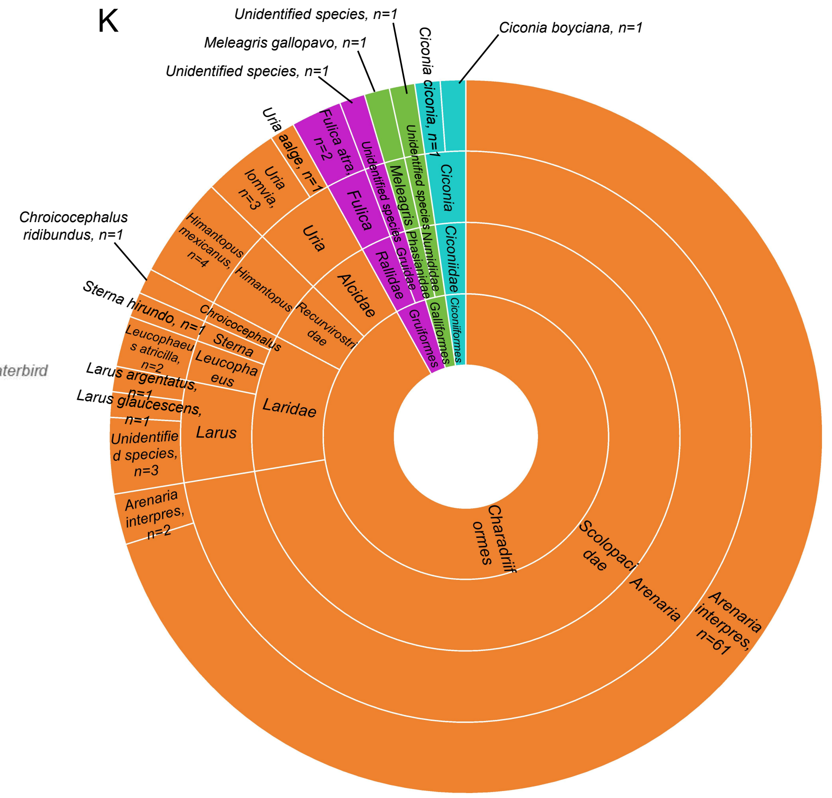

Unidentified species, n=1
Meleagris gallopavo, n=1
Unidentified species, n=1
Ciconia ciconia, n=1
Ciconia boyciana, n=1
Uria aalge, n=1
Fulica atra n=2
Uria lomvia, n=3
Chroicocephalus ridibundus, n=1
Himantopus mexicanus, n=4
Sterna hirundo, n=1
Leucophaeus atricilla, n=2
Larus argentatus, n=1
Larus glaucescens, n=1
Unidentified species, n=3
Arenaria interpres, n=2
Arenaria interpres, n=61
Uria
Fulica
Unidentified species
Meleagris
Unidentified species
Ciconia
Himantopus
Chroicocephalus
Sterna
Leucophaeus
Larus
Alcidae
Rallidae
Phasianidae
Numididae
Gruidae
Ciconiidae
Recurvirostridae
Laridae
Scolopacidae
Arenaria
Gruiformes
Galliformes
Ciconiiformes
Charadriiformes

L

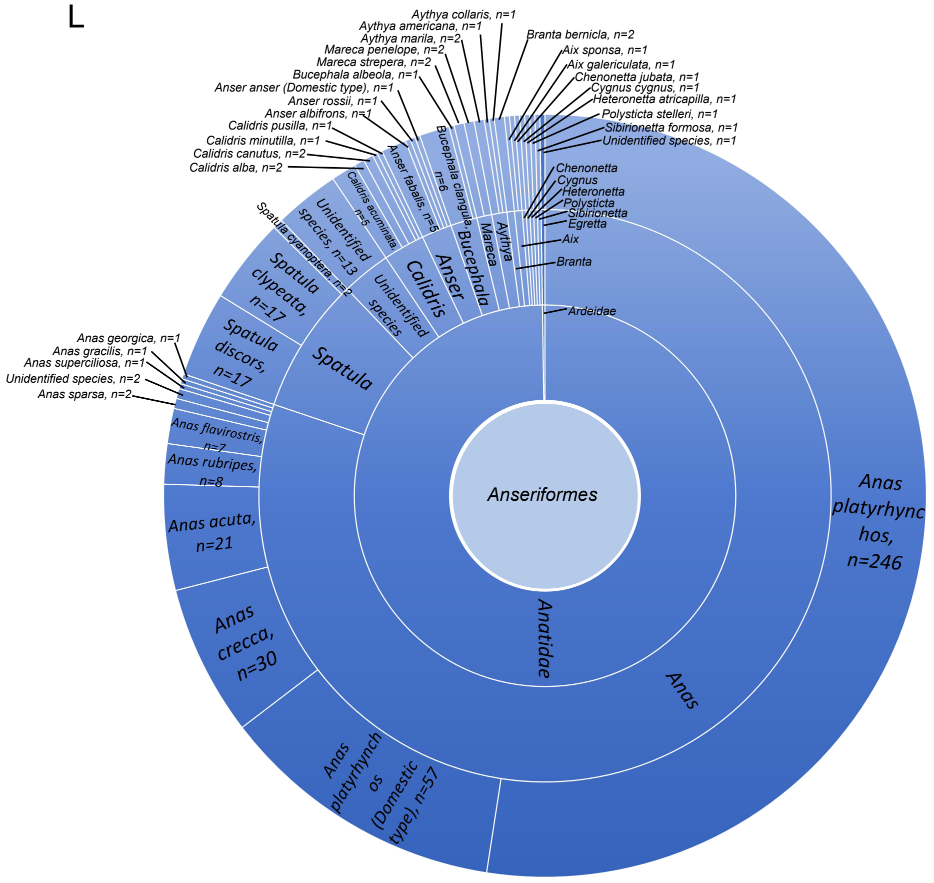

Aythya collaris, n=1
Aythya americana, n=1
Aythya marila, n=2
Mareca penelope, n=2
Mareca strepera, n=2
Bucephala albeola, n=1
Anser anser (Domestic type), n=1
Anser rossii, n=1
Anser albifrons, n=1
Calidris pusilla, n=1
Calidris minutilla, n=1
Calidris canutus, n=2
Calidris alba, n=2
Branta bernicla, n=2
Aix sponsa, n=1
Aix galericulata, n=1
Chenonetta jubata, n=1
Cygnus cygnus, n=1
Heteronetta atricapilla, n=1
Polysticta stelleri, n=1
Sibirionetta formosa, n=1
Unidentified species, n=1
Chenonetta
Cygnus
Heteronetta
Polysticta
Sibirionetta
Egretta
Aix
Branta
Ardeidae
Bucephala clangula n=6
Anser fabalis, n=5
Calidris acuminata n=5
Unidentified species, n=13
Spatula cyanoptera, n=2
Spatula clypeata, n=17
Spatula discors, n=17
Anas georgica, n=1
Anas gracilis, n=1
Anas superciliosa, n=1
Unidentified species, n=2
Anas sparsa, n=2
Anas flavirostris, n=7
Anas rubripes, n=8
Anas acuta, n=21
Anas crecca, n=30
Anas platyrhynchos (Domestic type), n=57
Anas platyrhynchos, n=246
Aythya
Mareca
Bucephala
Anser
Calidris
Unidentified species
Spatula
Anas
Anatidae
Anseriformes

Figure S2

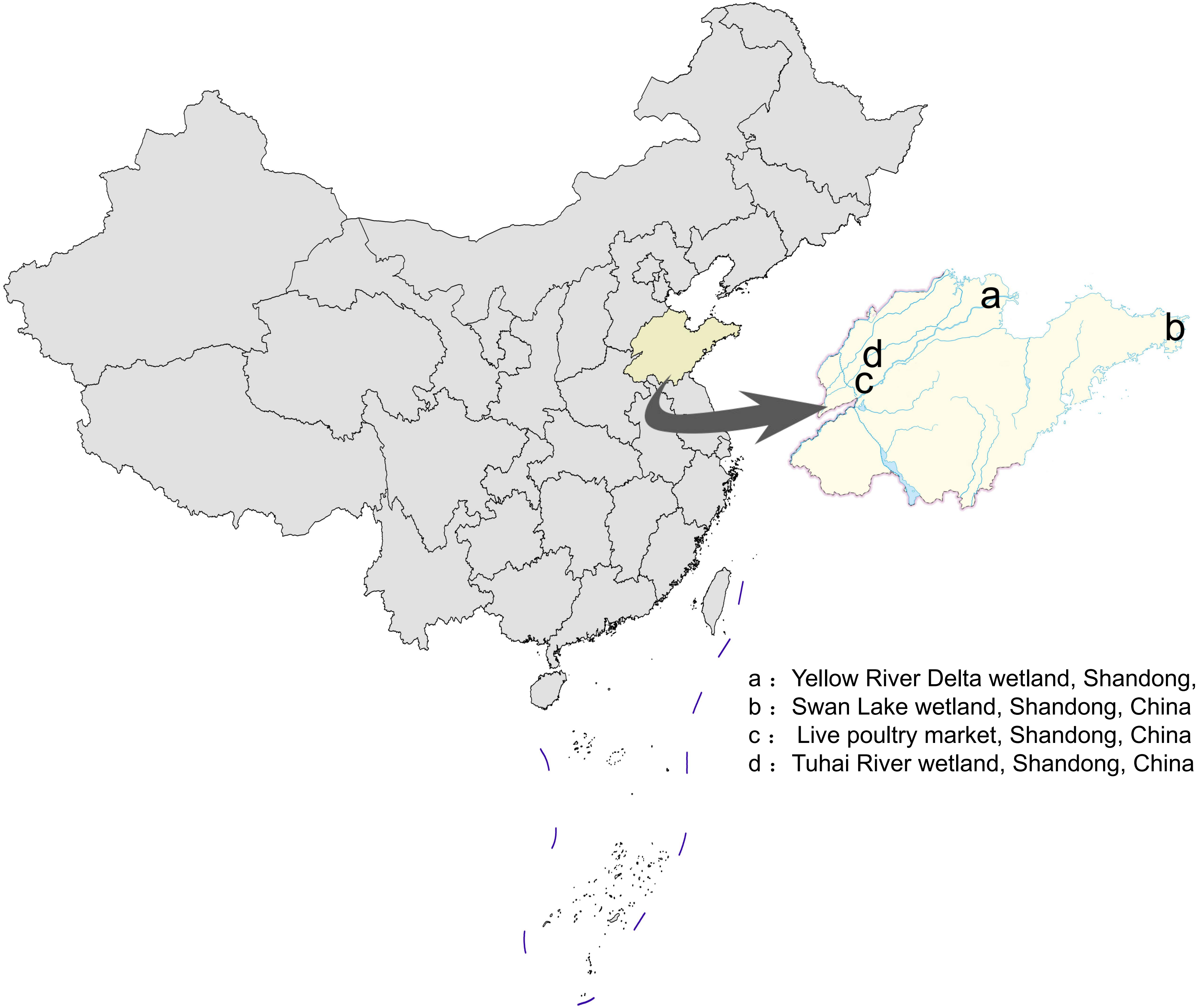

a： Yellow River Delta wetland, Shandong, China
b： Swan Lake wetland, Shandong, China
c： Live poultry market, Shandong, China
d： Tuhai River wetland, Shandong, China

Figure S3

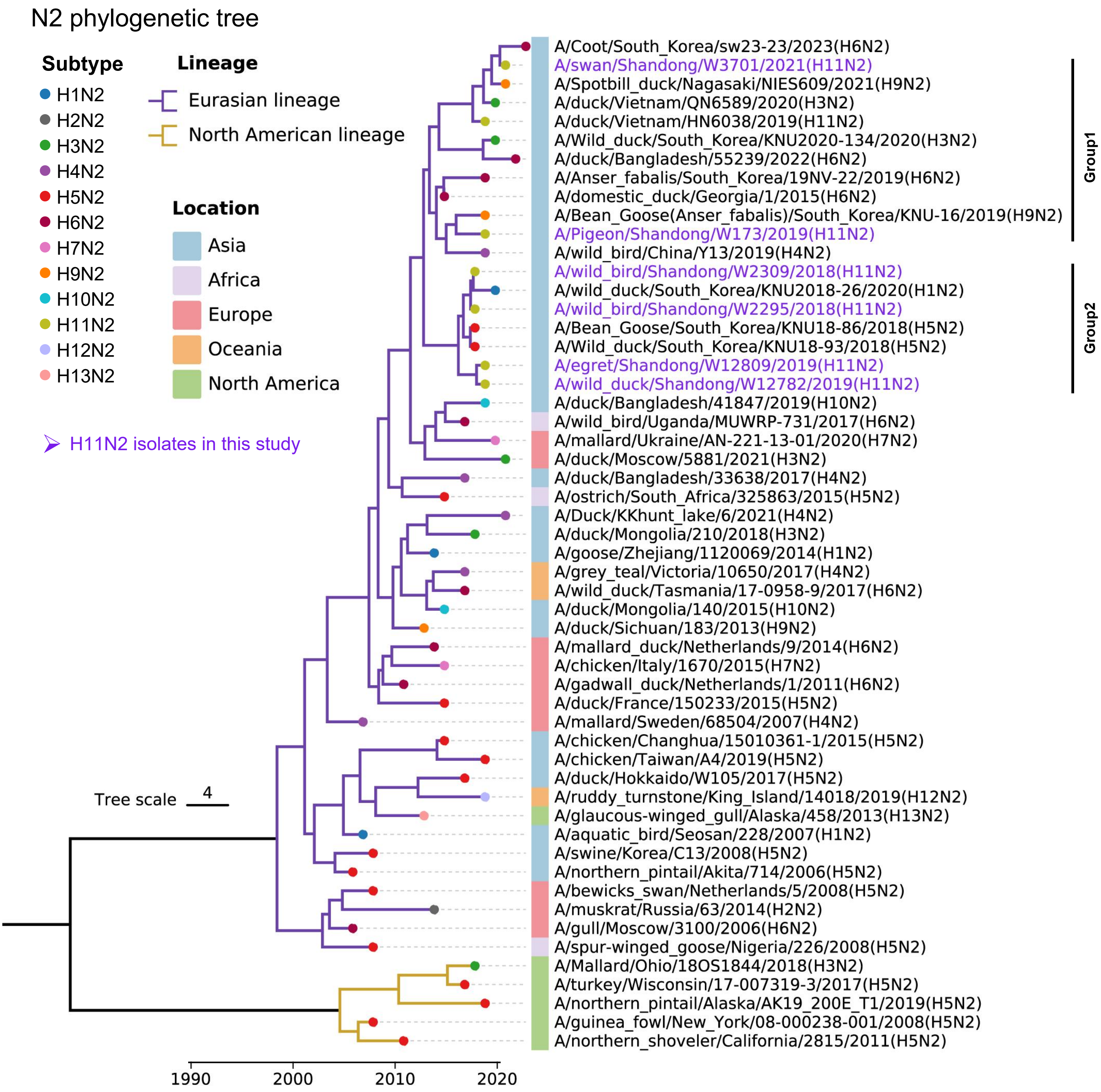
N2 phylogenetic tree
Subtype
H1N2
H2N2
H3N2
H4N2
H5N2
H6N2
H7N2
H9N2
H10N2
H11N2
H12N2
H13N2
Lineage
Eurasian lineage
North American lineage
Location
Asia
Africa
Europe
Oceania
North America
H11N2 isolates in this study
Tree scale 4
1990
2000
2010
2020
A/Coot/South_Korea/sw23-23/2023(H6N2)
A/swan/Shandong/W3701/2021(H11N2)
A/Spotbill_duck/Nagasaki/NIES609/2021(H9N2)
A/duck/Vietnam/QN6589/2020(H3N2)
A/duck/Vietnam/HN6038/2019(H11N2)
A/Wild_duck/South_Korea/KNU2020-134/2020(H3N2)
A/duck/Bangladesh/55239/2022(H6N2)
A/Anser_fabalis/South_Korea/19NV-22/2019(H6N2)
A/domestic_duck/Georgia/1/2015(H6N2)
A/Bean_Goose(Anser_fabalis)/South_Korea/KNU-16/2019(H9N2)
A/Pigeon/Shandong/W173/2019(H11N2)
A/wild_bird/China/Y13/2019(H4N2)
A/wild_bird/Shandong/W2309/2018(H11N2)
A/wild_duck/South_Korea/KNU2018-26/2020(H1N2)
A/wild_bird/Shandong/W2295/2018(H11N2)
A/Bean_Goose/South_Korea/KNU18-86/2018(H5N2)
A/Wild_duck/South_Korea/KNU18-93/2018(H5N2)
A/egret/Shandong/W12809/2019(H11N2)
A/wild_duck/Shandong/W12782/2019(H11N2)
A/duck/Bangladesh/41847/2019(H10N2)
A/wild_bird/Uganda/MUWRP-731/2017(H6N2)
A/mallard/Ukraine/AN-221-13-01/2020(H7N2)
A/duck/Moscow/5881/2021(H3N2)
A/duck/Bangladesh/33638/2017(H4N2)
A/ostrich/South_Africa/325863/2015(H5N2)
A/Duck/KKhunt_lake/6/2021(H4N2)
A/duck/Mongolia/210/2018(H3N2)
A/goose/Zhejiang/1120069/2014(H1N2)
A/grey_teal/Victoria/10650/2017(H4N2)
A/wild_duck/Tasmania/17-0958-9/2017(H6N2)
A/duck/Mongolia/140/2015(H10N2)
A/duck/Sichuan/183/2013(H9N2)
A/mallard_duck/Netherlands/9/2014(H6N2)
A/chicken/Italy/1670/2015(H7N2)
A/gadwall_duck/Netherlands/1/2011(H6N2)
A/duck/France/150233/2015(H5N2)
A/mallard/Sweden/68504/2007(H4N2)
A/chicken/Changhua/15010361-1/2015(H5N2)
A/chicken/Taiwan/A4/2019(H5N2)
A/duck/Hokkaido/W105/2017(H5N2)
A/ruddy_turnstone/King_Island/14018/2019(H12N2)
A/glaucous-winged_gull/Alaska/458/2013(H13N2)
A/aquatic_bird/Seosan/228/2007(H1N2)
A/swine/Korea/C13/2008(H5N2)
A/northern_pintail/Akita/714/2006(H5N2)
A/bewicks_swan/Netherlands/5/2008(H5N2)
A/muskrat/Russia/63/2014(H2N2)
A/gull/Moscow/3100/2006(H6N2)
A/spur-winged_goose/Nigeria/226/2008(H5N2)
A/Mallard/Ohio/18OS1844/2018(H3N2)
A/turkey/Wisconsin/17-007319-3/2017(H5N2)
A/northern_pintail/Alaska/AK19_200E_T1/2019(H5N2)
A/guinea_fowl/New_York/08-000238-001/2008(H5N2)
A/northern_shoveler/California/2815/2011(H5N2)
Group1
Group2

N3 phylogenetic tree

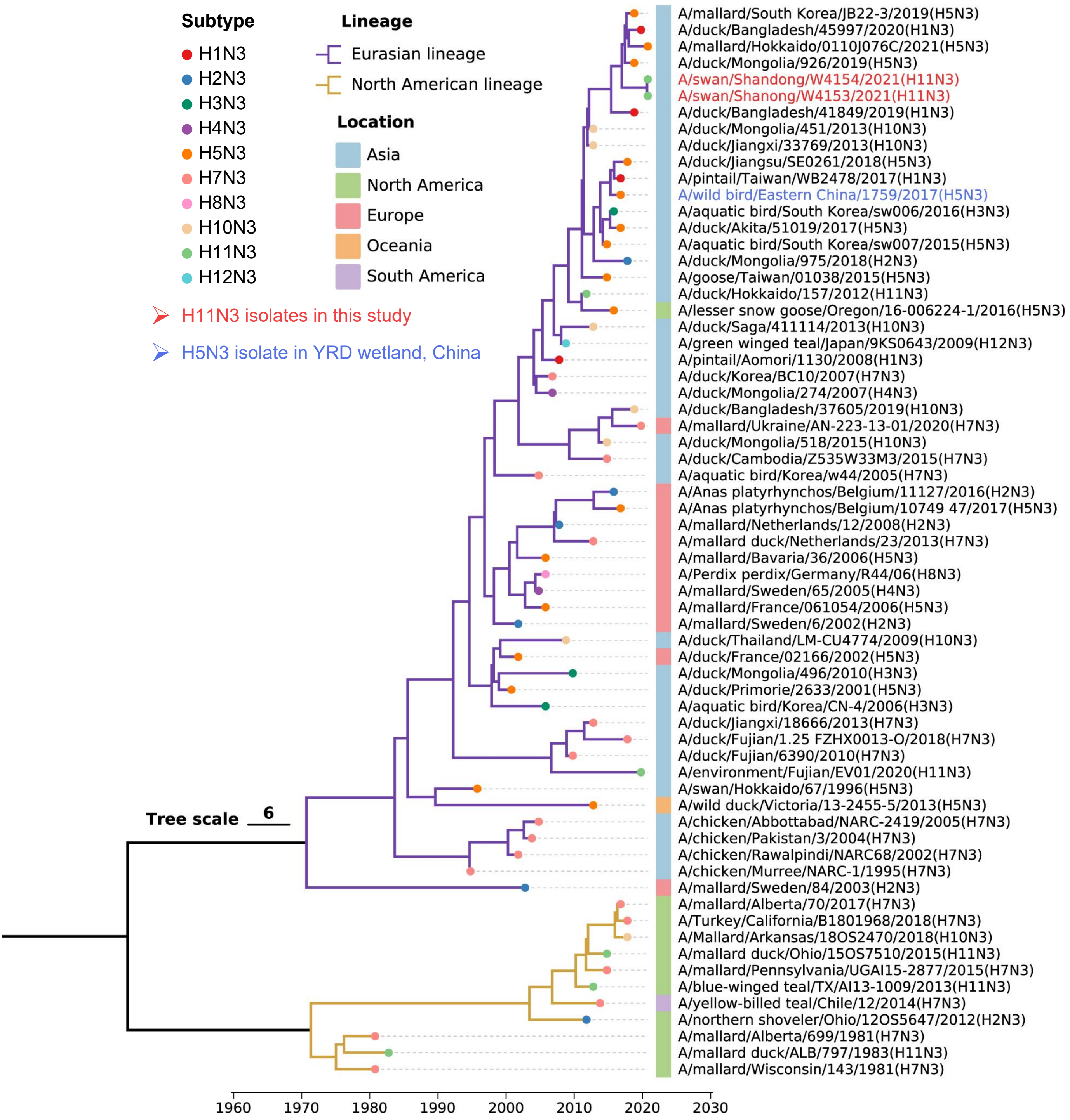

Figure S4

PB2

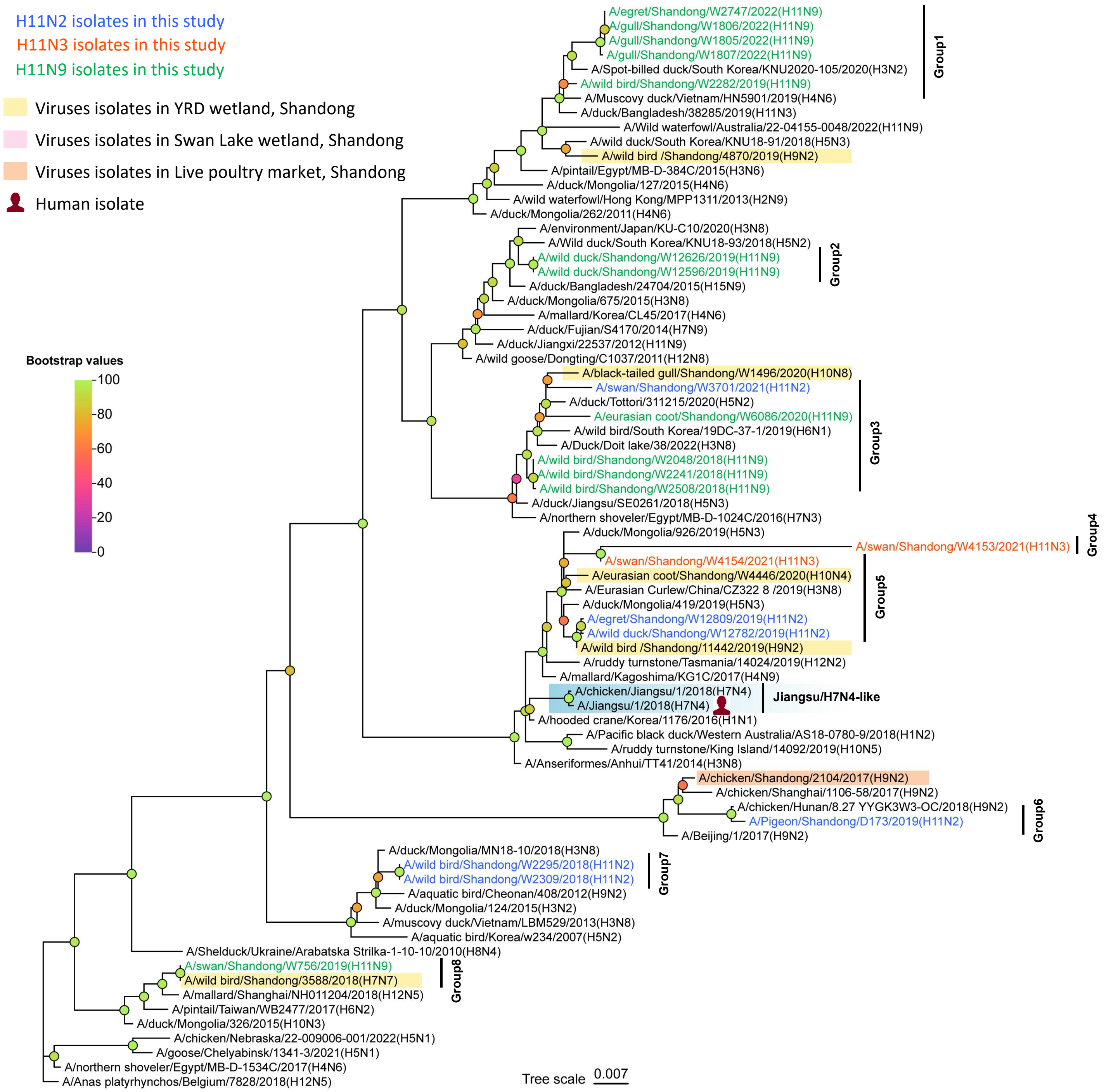

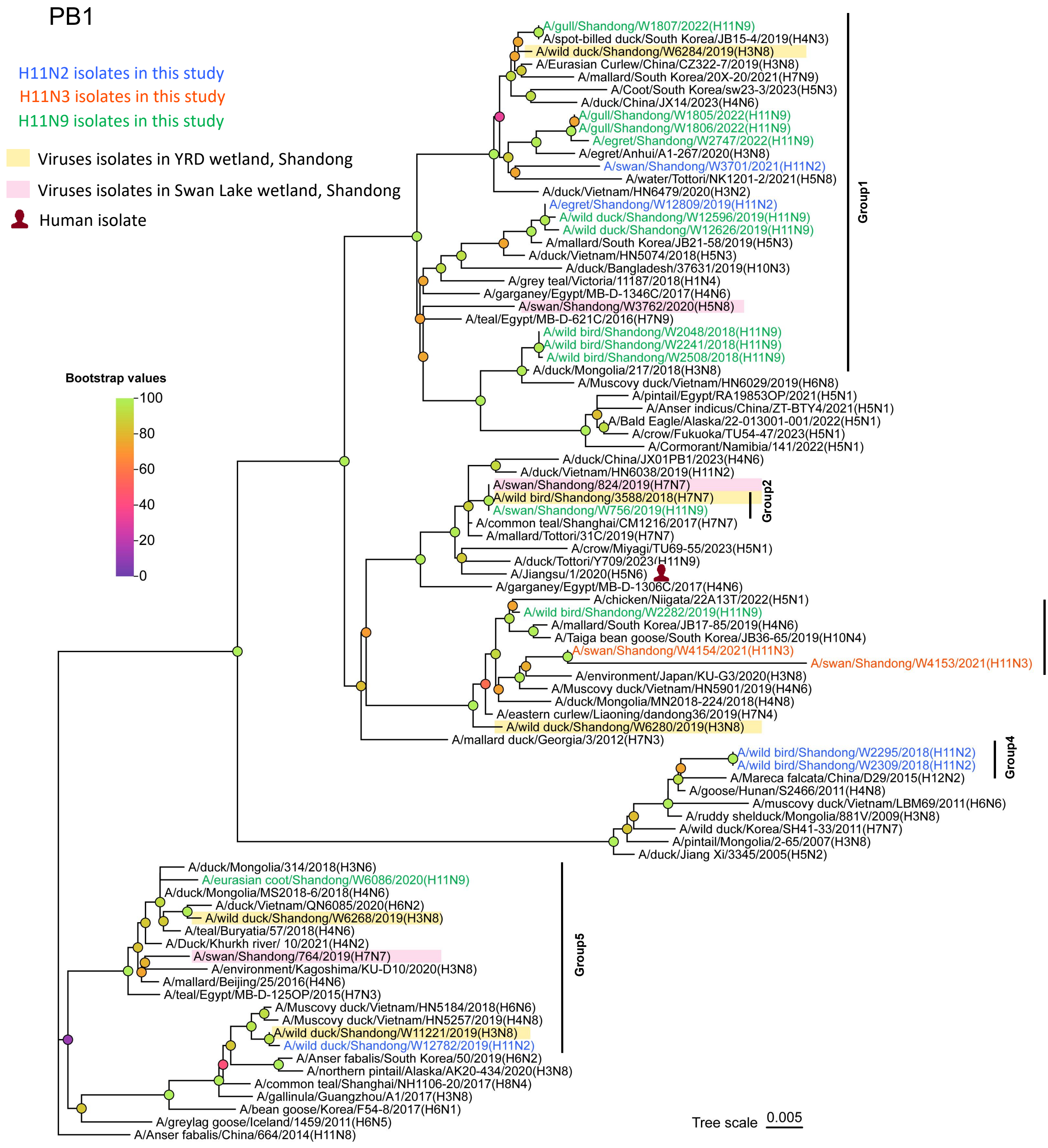

PB1
H11N2 isolates in this study
H11N3 isolates in this study
H11N9 isolates in this study
Viruses isolates in YRD wetland, Shandong
Viruses isolates in Swan Lake wetland, Shandong
Human isolate
Bootstrap values
100
80
60
40
20
0
A/gull/Shandong/W1807/2022(H11N9)
A/spot-billed duck/South Korea/JB15-4/2019(H4N3)
A/wild duck/Shandong/W6284/2019(H3N8)
A/Eurasian Curlew/China/CZ322-7/2019(H3N8)
A/mallard/South Korea/20X-20/2021(H7N9)
A/Coot/South Korea/sw23-3/2023(H5N3)
A/duck/China/JX14/2023(H4N6)
A/gull/Shandong/W1805/2022(H11N9)
A/gull/Shandong/W1806/2022(H11N9)
A/egret/Shandong/W2747/2022(H11N9)
A/egret/Anhui/A1-267/2020(H3N8)
A/swan/Shandong/W3701/2021(H11N2)
A/water/Tottori/NK1201-2/2021(H5N8)
A/duck/Vietnam/HN6479/2020(H3N2)
A/egret/Shandong/W12809/2019(H11N2)
A/wild duck/Shandong/W12596/2019(H11N9)
A/wild duck/Shandong/W12626/2019(H11N9)
A/mallard/South Korea/JB21-58/2019(H5N3)
A/duck/Vietnam/HN5074/2018(H5N3)
A/duck/Bangladesh/37631/2019(H10N3)
A/grey teal/Victoria/11187/2018(H1N4)
A/garganey/Egypt/MB-D-1346C/2017(H4N6)
A/swan/Shandong/W3762/2020(H5N8)
A/teal/Egypt/MB-D-621C/2016(H7N9)
A/wild bird/Shandong/W2048/2018(H11N9)
A/wild bird/Shandong/W2241/2018(H11N9)
A/wild bird/Shandong/W2508/2018(H11N9)
A/duck/Mongolia/217/2018(H3N8)
A/Muscovy duck/Vietnam/HN6029/2019(H6N8)
A/pintail/Egypt/RA19853OP/2021(H5N1)
A/Anser indicus/China/ZT-BTY4/2021(H5N1)
A/Bald Eagle/Alaska/22-013001-001/2022(H5N1)
A/crow/Fukuoka/TU54-47/2023(H5N1)
A/Cormorant/Namibia/141/2022(H5N1)
Group1
A/duck/China/JX01PB1/2023(H4N6)
A/duck/Vietnam/HN6038/2019(H11N2)
A/swan/Shandong/824/2019(H7N7)
A/wild bird/Shandong/3588/2018(H7N7)
A/swan/Shandong/W756/2019(H11N9)
A/common teal/Shanghai/CM1216/2017(H7N7)
A/mallard/Tottori/31C/2019(H7N7)
A/crow/Miyagi/TU69-55/2023(H5N1)
A/duck/Tottori/Y709/2023(H11N9)
A/Jiangsu/1/2020(H5N6)
A/garganey/Egypt/MB-D-1306C/2017(H4N6)
Group2
A/chicken/Niigata/22A13T/2022(H5N1)
A/wild bird/Shandong/W2282/2019(H11N9)
A/mallard/South Korea/JB17-85/2019(H4N6)
A/Taiga bean goose/South Korea/JB36-65/2019(H10N4)
A/swan/Shandong/W4154/2021(H11N3)
A/swan/Shandong/W4153/2021(H11N3)
A/environment/Japan/KU-G3/2020(H3N8)
A/Muscovy duck/Vietnam/HN5901/2019(H4N6)
A/duck/Mongolia/MN2018-224/2018(H4N8)
A/eastern curlew/Liaoning/dandong36/2019(H7N4)
A/wild duck/Shandong/W6280/2019(H3N8)
A/mallard duck/Georgia/3/2012(H7N3)
Group3
A/wild bird/Shandong/W2295/2018(H11N2)
A/wild bird/Shandong/W2309/2018(H11N2)
A/Mareca falcata/China/D29/2015(H12N2)
A/goose/Hunan/S2466/2011(H4N8)
A/muscovy duck/Vietnam/LBM69/2011(H6N6)
A/ruddy shelduck/Mongolia/881V/2009(H3N8)
A/wild duck/Korea/SH41-33/2011(H7N7)
A/pintail/Mongolia/2-65/2007(H3N8)
A/duck/Jiang Xi/3345/2005(H5N2)
Group4
A/duck/Mongolia/314/2018(H3N6)
A/eurasian coot/Shandong/W6086/2020(H11N9)
A/duck/Mongolia/MS2018-6/2018(H4N6)
A/duck/Vietnam/QN6085/2020(H6N2)
A/wild duck/Shandong/W6268/2019(H3N8)
A/teal/Buryatia/57/2018(H4N6)
A/Duck/Khurkh river/ 10/2021(H4N2)
A/swan/Shandong/764/2019(H7N7)
A/environment/Kagoshima/KU-D10/2020(H3N8)
A/mallard/Beijing/25/2016(H4N6)
A/teal/Egypt/MB-D-125OP/2015(H7N3)
A/Muscovy duck/Vietnam/HN5184/2018(H6N6)
A/Muscovy duck/Vietnam/HN5257/2019(H4N8)
A/wild duck/Shandong/W11221/2019(H3N8)
A/wild duck/Shandong/W12782/2019(H11N2)
A/Anser fabalis/South Korea/50/2019(H6N2)
A/northern pintail/Alaska/AK20-434/2020(H3N8)
A/common teal/Shanghai/NH1106-20/2017(H8N4)
A/gallinula/Guangzhou/A1/2017(H3N8)
A/bean goose/Korea/F54-8/2017(H6N1)
A/greylag goose/Iceland/1459/2011(H6N5)
A/Anser fabalis/China/664/2014(H11N8)
Group5
Tree scale 0.005

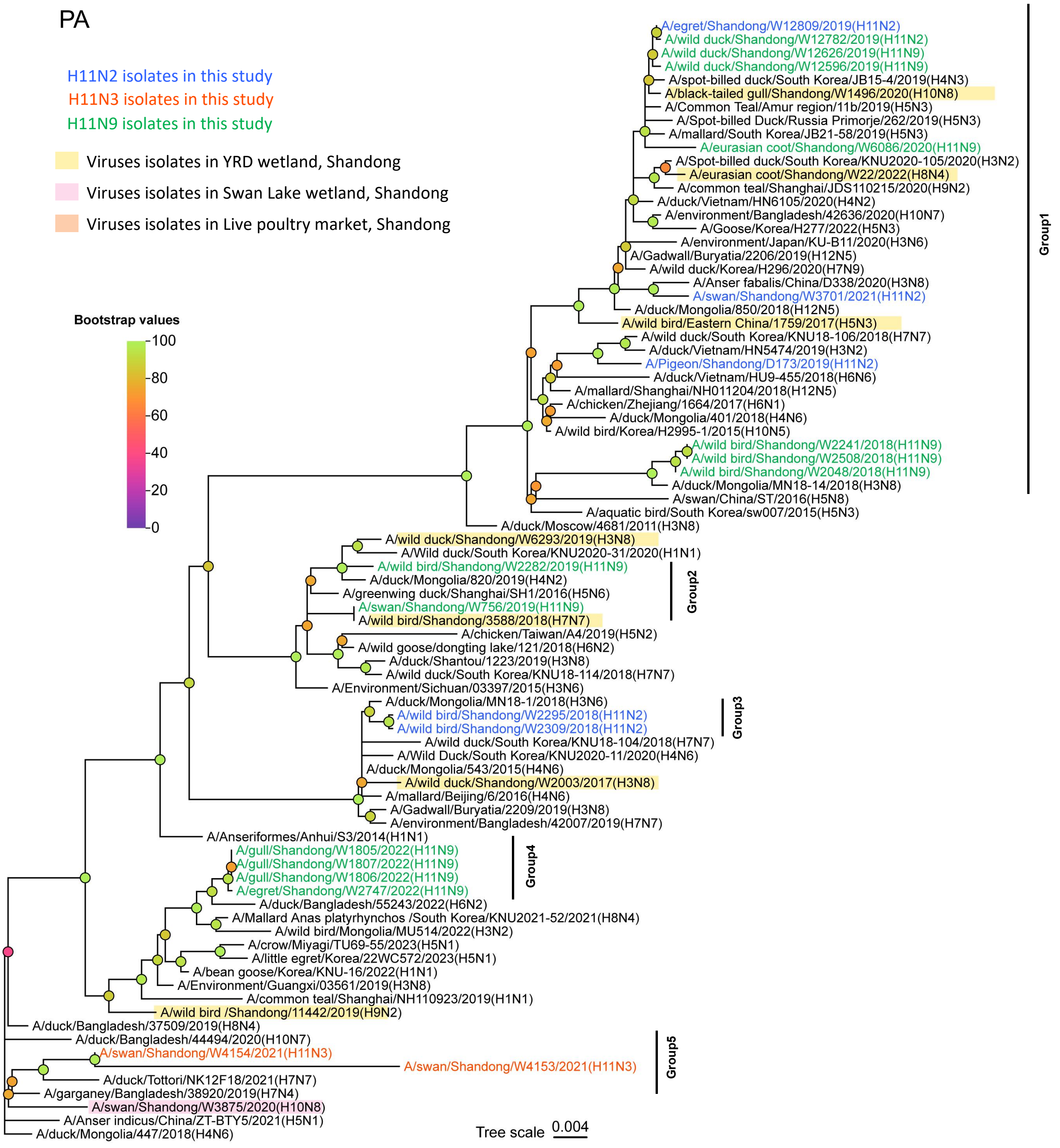
PA
H11N2 isolates in this study
H11N3 isolates in this study
H11N9 isolates in this study
Viruses isolates in YRD wetland, Shandong
Viruses isolates in Swan Lake wetland, Shandong
Viruses isolates in Live poultry market, Shandong
Bootstrap values
100
80
60
40
20
0
A/egret/Shandong/W12809/2019(H11N2)
A/wild duck/Shandong/W12782/2019(H11N2)
A/wild duck/Shandong/W12626/2019(H11N9)
A/wild duck/Shandong/W12596/2019(H11N9)
A/spot-billed duck/South Korea/JB15-4/2019(H4N3)
A/black-tailed gull/Shandong/W1496/2020(H10N8)
A/Common Teal/Amur region/11b/2019(H5N3)
A/Spot-billed Duck/Russia Primorje/262/2019(H5N3)
A/mallard/South Korea/JB21-58/2019(H5N3)
A/eurasian coot/Shandong/W6086/2020(H11N9)
A/Spot-billed duck/South Korea/KNU2020-105/2020(H3N2)
A/eurasian coot/Shandong/W22/2022(H8N4)
A/common teal/Shanghai/JDS110215/2020(H9N2)
A/duck/Vietnam/HN6105/2020(H4N2)
A/environment/Bangladesh/42636/2020(H10N7)
A/Goose/Korea/H277/2022(H5N3)
A/environment/Japan/KU-B11/2020(H3N6)
A/Gadwall/Buryatia/2206/2019(H12N5)
A/wild duck/Korea/H296/2020(H7N9)
A/Anser fabalis/China/D338/2020(H3N8)
A/swan/Shandong/W3701/2021(H11N2)
A/duck/Mongolia/850/2018(H12N5)
A/wild bird/Eastern China/1759/2017(H5N3)
A/wild duck/South Korea/KNU18-106/2018(H7N7)
A/duck/Vietnam/HN5474/2019(H3N2)
A/Pigeon/Shandong/D173/2019(H11N2)
A/duck/Vietnam/HU9-455/2018(H6N6)
A/mallard/Shanghai/NH011204/2018(H12N5)
A/chicken/Zhejiang/1664/2017(H6N1)
A/duck/Mongolia/401/2018(H4N6)
A/wild bird/Korea/H2995-1/2015(H10N5)
A/wild bird/Shandong/W2241/2018(H11N9)
A/wild bird/Shandong/W2508/2018(H11N9)
A/wild bird/Shandong/W2048/2018(H11N9)
A/duck/Mongolia/MN18-14/2018(H3N8)
A/swan/China/ST/2016(H5N8)
A/aquatic bird/South Korea/sw007/2015(H5N3)
A/duck/Moscow/4681/2011(H3N8)
Group1
A/wild duck/Shandong/W6293/2019(H3N8)
A/Wild duck/South Korea/KNU2020-31/2020(H1N1)
A/wild bird/Shandong/W2282/2019(H11N9)
A/duck/Mongolia/820/2019(H4N2)
A/greenwing duck/Shanghai/SH1/2016(H5N6)
A/swan/Shandong/W756/2019(H11N9)
A/wild bird/Shandong/3588/2018(H7N7)
Group2
A/chicken/Taiwan/A4/2019(H5N2)
A/wild goose/dongting lake/121/2018(H6N2)
A/duck/Shantou/1223/2019(H3N8)
A/wild duck/South Korea/KNU18-114/2018(H7N7)
A/Environment/Sichuan/03397/2015(H3N6)
A/duck/Mongolia/MN18-1/2018(H3N6)
A/wild bird/Shandong/W2295/2018(H11N2)
A/wild bird/Shandong/W2309/2018(H11N2)
Group3
A/wild duck/South Korea/KNU18-104/2018(H7N7)
A/Wild Duck/South Korea/KNU2020-11/2020(H4N6)
A/duck/Mongolia/543/2015(H4N6)
A/wild duck/Shandong/W2003/2017(H3N8)
A/mallard/Beijing/6/2016(H4N6)
A/Gadwall/Buryatia/2209/2019(H3N8)
A/environment/Bangladesh/42007/2019(H7N7)
A/Anseriformes/Anhui/S3/2014(H1N1)
A/gull/Shandong/W1805/2022(H11N9)
A/gull/Shandong/W1807/2022(H11N9)
A/gull/Shandong/W1806/2022(H11N9)
A/egret/Shandong/W2747/2022(H11N9)
Group4
A/duck/Bangladesh/55243/2022(H6N2)
A/Mallard Anas platyrhynchos /South Korea/KNU2021-52/2021(H8N4)
A/wild bird/Mongolia/MU514/2022(H3N2)
A/crow/Miyagi/TU69-55/2023(H5N1)
A/little egret/Korea/22WC572/2023(H5N1)
A/bean goose/Korea/KNU-16/2022(H1N1)
A/Environment/Guangxi/03561/2019(H3N8)
A/common teal/Shanghai/NH110923/2019(H1N1)
A/wild bird /Shandong/11442/2019(H9N2)
A/duck/Bangladesh/37509/2019(H8N4)
A/duck/Bangladesh/44494/2020(H10N7)
A/swan/Shandong/W4154/2021(H11N3)
A/swan/Shandong/W4153/2021(H11N3)
Group5
A/duck/Tottori/NK12F18/2021(H7N7)
A/garganey/Bangladesh/38920/2019(H7N4)
A/swan/Shandong/W3875/2020(H10N8)
A/Anser indicus/China/ZT-BTY5/2021(H5N1)
A/duck/Mongolia/447/2018(H4N6)
Tree scale 0.004

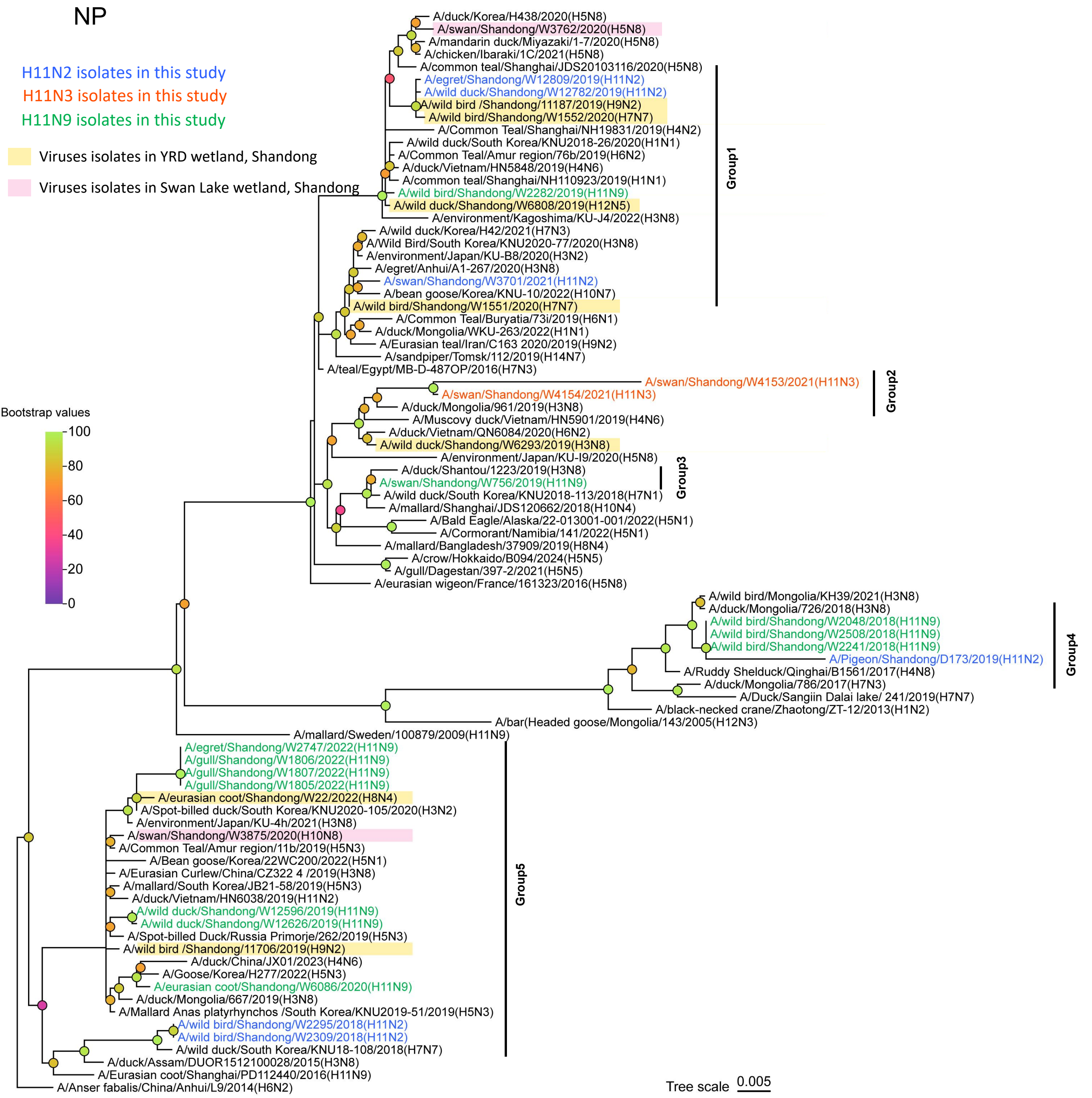
NP
H11N2 isolates in this study
H11N3 isolates in this study
H11N9 isolates in this study
Viruses isolates in YRD wetland, Shandong
Viruses isolates in Swan Lake wetland, Shandong
Bootstrap values
100
80
60
40
20
0
A/duck/Korea/H438/2020(H5N8)
A/swan/Shandong/W3762/2020(H5N8)
A/mandarin duck/Miyazaki/1-7/2020(H5N8)
A/chicken/Ibaraki/1C/2021(H5N8)
A/common teal/Shanghai/JDS20103116/2020(H5N8)
A/egret/Shandong/W12809/2019(H11N2)
A/wild duck/Shandong/W12782/2019(H11N2)
A/wild bird /Shandong/11187/2019(H9N2)
A/wild bird/Shandong/W1552/2020(H7N7)
A/Common Teal/Shanghai/NH19831/2019(H4N2)
A/wild duck/South Korea/KNU2018-26/2020(H1N1)
A/Common Teal/Amur region/76b/2019(H6N2)
A/duck/Vietnam/HN5848/2019(H4N6)
A/common teal/Shanghai/NH110923/2019(H1N1)
A/wild bird/Shandong/W2282/2019(H11N9)
A/wild duck/Shandong/W6808/2019(H12N5)
A/environment/Kagoshima/KU-J4/2022(H3N8)
A/wild duck/Korea/H42/2021(H7N3)
A/Wild Bird/South Korea/KNU2020-77/2020(H3N8)
A/environment/Japan/KU-B8/2020(H3N2)
A/egret/Anhui/A1-267/2020(H3N8)
A/swan/Shandong/W3701/2021(H11N2)
A/bean goose/Korea/KNU-10/2022(H10N7)
A/wild bird/Shandong/W1551/2020(H7N7)
A/Common Teal/Buryatia/73i/2019(H6N1)
A/duck/Mongolia/WKU-263/2022(H1N1)
A/Eurasian teal/Iran/C163 2020/2019(H9N2)
A/sandpiper/Tomsk/112/2019(H14N7)
A/teal/Egypt/MB-D-487OP/2016(H7N3)
Group1
A/swan/Shandong/W4153/2021(H11N3)
A/swan/Shandong/W4154/2021(H11N3)
A/duck/Mongolia/961/2019(H3N8)
A/Muscovy duck/Vietnam/HN5901/2019(H4N6)
A/duck/Vietnam/QN6084/2020(H6N2)
A/wild duck/Shandong/W6293/2019(H3N8)
A/environment/Japan/KU-I9/2020(H5N8)
Group2
A/duck/Shantou/1223/2019(H3N8)
A/swan/Shandong/W756/2019(H11N9)
A/wild duck/South Korea/KNU2018-113/2018(H7N1)
A/mallard/Shanghai/JDS120662/2018(H10N4)
Group3
A/Bald Eagle/Alaska/22-013001-001/2022(H5N1)
A/Cormorant/Namibia/141/2022(H5N1)
A/mallard/Bangladesh/37909/2019(H8N4)
A/crow/Hokkaido/B094/2024(H5N5)
A/gull/Dagestan/397-2/2021(H5N5)
A/eurasian wigeon/France/161323/2016(H5N8)
A/wild bird/Mongolia/KH39/2021(H3N8)
A/duck/Mongolia/726/2018(H3N8)
A/wild bird/Shandong/W2048/2018(H11N9)
A/wild bird/Shandong/W2508/2018(H11N9)
A/wild bird/Shandong/W2241/2018(H11N9)
A/Pigeon/Shandong/D173/2019(H11N2)
A/Ruddy Shelduck/Qinghai/B1561/2017(H4N8)
A/duck/Mongolia/786/2017(H7N3)
A/Duck/Sangiin Dalai lake/ 241/2019(H7N7)
A/black-necked crane/Zhaotong/ZT-12/2013(H1N2)
Group4
A/bar(Headed goose/Mongolia/143/2005(H12N3)
A/mallard/Sweden/100879/2009(H11N9)
A/egret/Shandong/W2747/2022(H11N9)
A/gull/Shandong/W1806/2022(H11N9)
A/gull/Shandong/W1807/2022(H11N9)
A/gull/Shandong/W1805/2022(H11N9)
A/eurasian coot/Shandong/W22/2022(H8N4)
A/Spot-billed duck/South Korea/KNU2020-105/2020(H3N2)
A/environment/Japan/KU-4h/2021(H3N8)
A/swan/Shandong/W3875/2020(H10N8)
A/Common Teal/Amur region/11b/2019(H5N3)
A/Bean goose/Korea/22WC200/2022(H5N1)
A/Eurasian Curlew/China/CZ322 4 /2019(H3N8)
A/mallard/South Korea/JB21-58/2019(H5N3)
A/duck/Vietnam/HN6038/2019(H11N2)
A/wild duck/Shandong/W12596/2019(H11N9)
A/wild duck/Shandong/W12626/2019(H11N9)
A/Spot-billed Duck/Russia Primorje/262/2019(H5N3)
A/wild bird /Shandong/11706/2019(H9N2)
A/duck/China/JX01/2023(H4N6)
A/Goose/Korea/H277/2022(H5N3)
A/eurasian coot/Shandong/W6086/2020(H11N9)
A/duck/Mongolia/667/2019(H3N8)
A/Mallard Anas platyrhynchos /South Korea/KNU2019-51/2019(H5N3)
A/wild bird/Shandong/W2295/2018(H11N2)
A/wild bird/Shandong/W2309/2018(H11N2)
A/wild duck/South Korea/KNU18-108/2018(H7N7)
A/duck/Assam/DUOR1512100028/2015(H3N8)
A/Eurasian coot/Shanghai/PD112440/2016(H11N9)
A/Anser fabalis/China/Anhui/L9/2014(H6N2)
Group5
Tree scale 0.005

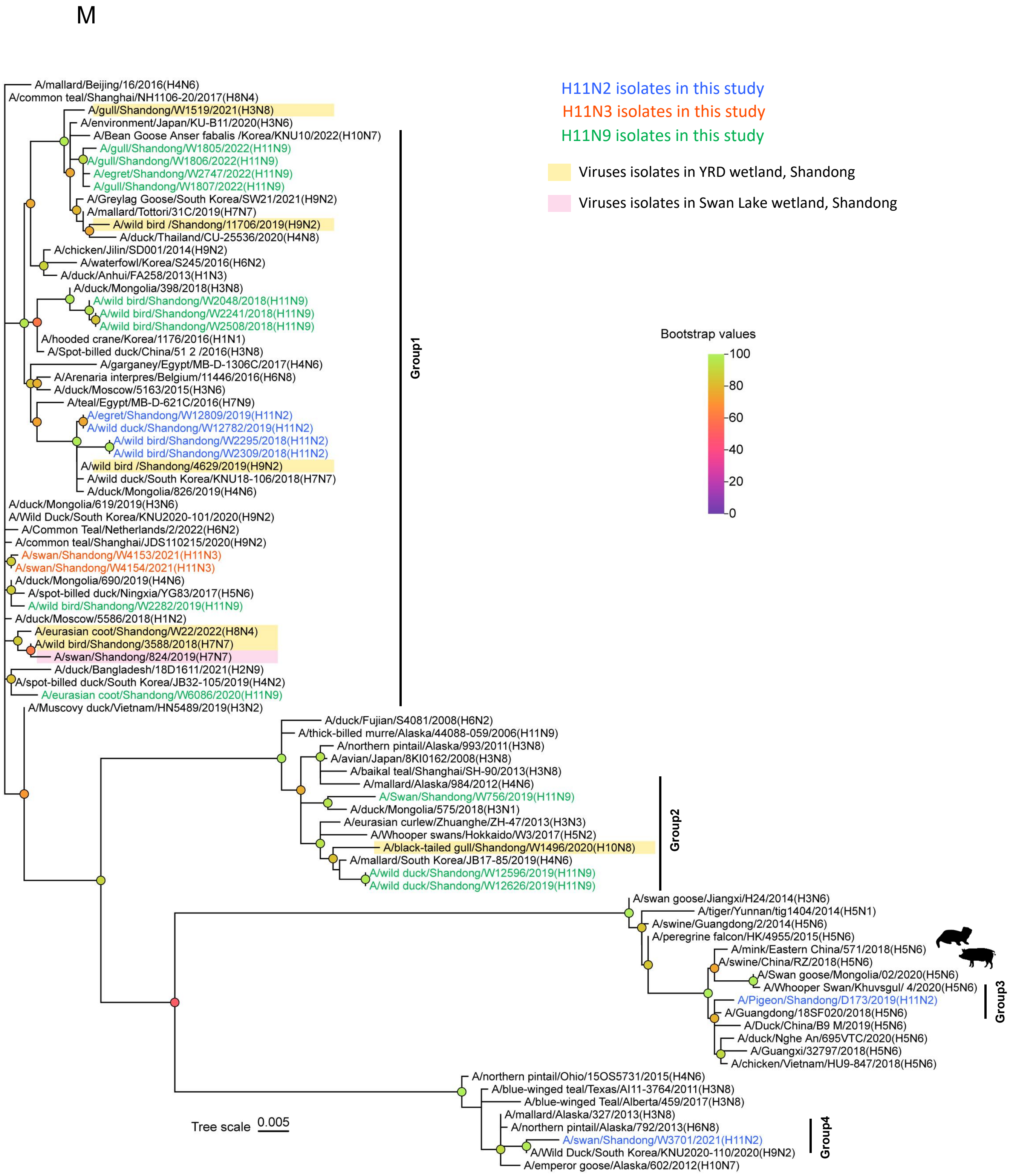
M
A/mallard/Beijing/16/2016(H4N6)
A/common teal/Shanghai/NH1106-20/2017(H8N4)
A/gull/Shandong/W1519/2021(H3N8)
A/environment/Japan/KU-B11/2020(H3N6)
A/Bean Goose Anser fabalis /Korea/KNU10/2022(H10N7)
A/gull/Shandong/W1805/2022(H11N9)
A/gull/Shandong/W1806/2022(H11N9)
A/egret/Shandong/W2747/2022(H11N9)
A/gull/Shandong/W1807/2022(H11N9)
A/Greylag Goose/South Korea/SW21/2021(H9N2)
A/mallard/Tottori/31C/2019(H7N7)
A/wild bird /Shandong/11706/2019(H9N2)
A/duck/Thailand/CU-25536/2020(H4N8)
A/chicken/Jilin/SD001/2014(H9N2)
A/waterfowl/Korea/S245/2016(H6N2)
A/duck/Anhui/FA258/2013(H1N3)
A/duck/Mongolia/398/2018(H3N8)
A/wild bird/Shandong/W2048/2018(H11N9)
A/wild bird/Shandong/W2241/2018(H11N9)
A/wild bird/Shandong/W2508/2018(H11N9)
A/hooded crane/Korea/1176/2016(H1N1)
A/Spot-billed duck/China/51 2 /2016(H3N8)
A/garganey/Egypt/MB-D-1306C/2017(H4N6)
A/Arenaria interpres/Belgium/11446/2016(H6N8)
A/duck/Moscow/5163/2015(H3N6)
A/teal/Egypt/MB-D-621C/2016(H7N9)
A/egret/Shandong/W12809/2019(H11N2)
A/wild duck/Shandong/W12782/2019(H11N2)
A/wild bird/Shandong/W2295/2018(H11N2)
A/wild bird/Shandong/W2309/2018(H11N2)
A/wild bird /Shandong/4629/2019(H9N2)
A/wild duck/South Korea/KNU18-106/2018(H7N7)
A/duck/Mongolia/826/2019(H4N6)
A/duck/Mongolia/619/2019(H3N6)
A/Wild Duck/South Korea/KNU2020-101/2020(H9N2)
A/Common Teal/Netherlands/2/2022(H6N2)
A/common teal/Shanghai/JDS110215/2020(H9N2)
A/swan/Shandong/W4153/2021(H11N3)
A/swan/Shandong/W4154/2021(H11N3)
A/duck/Mongolia/690/2019(H4N6)
A/spot-billed duck/Ningxia/YG83/2017(H5N6)
A/wild bird/Shandong/W2282/2019(H11N9)
A/duck/Moscow/5586/2018(H1N2)
A/eurasian coot/Shandong/W22/2022(H8N4)
A/wild bird/Shandong/3588/2018(H7N7)
A/swan/Shandong/824/2019(H7N7)
A/duck/Bangladesh/18D1611/2021(H2N9)
A/spot-billed duck/South Korea/JB32-105/2019(H4N2)
A/eurasian coot/Shandong/W6086/2020(H11N9)
A/Muscovy duck/Vietnam/HN5489/2019(H3N2)
Group1
A/duck/Fujian/S4081/2008(H6N2)
A/thick-billed murre/Alaska/44088-059/2006(H11N9)
A/northern pintail/Alaska/993/2011(H3N8)
A/avian/Japan/8KI0162/2008(H3N8)
A/baikal teal/Shanghai/SH-90/2013(H3N8)
A/mallard/Alaska/984/2012(H4N6)
A/Swan/Shandong/W756/2019(H11N9)
A/duck/Mongolia/575/2018(H3N1)
A/eurasian curlew/Zhuanghe/ZH-47/2013(H3N3)
A/Whooper swans/Hokkaido/W3/2017(H5N2)
A/black-tailed gull/Shandong/W1496/2020(H10N8)
A/mallard/South Korea/JB17-85/2019(H4N6)
A/wild duck/Shandong/W12596/2019(H11N9)
A/wild duck/Shandong/W12626/2019(H11N9)
Group2
A/swan goose/Jiangxi/H24/2014(H3N6)
A/tiger/Yunnan/tig1404/2014(H5N1)
A/swine/Guangdong/2/2014(H5N6)
A/peregrine falcon/HK/4955/2015(H5N6)
A/mink/Eastern China/571/2018(H5N6)
A/swine/China/RZ/2018(H5N6)
A/Swan goose/Mongolia/02/2020(H5N6)
A/Whooper Swan/Khuvsgul/ 4/2020(H5N6)
A/Pigeon/Shandong/D173/2019(H11N2)
A/Guangdong/18SF020/2018(H5N6)
A/Duck/China/B9 M/2019(H5N6)
A/duck/Nghe An/695VTC/2020(H5N6)
A/Guangxi/32797/2018(H5N6)
A/chicken/Vietnam/HU9-847/2018(H5N6)
Group3
A/northern pintail/Ohio/15OS5731/2015(H4N6)
A/blue-winged teal/Texas/AI11-3764/2011(H3N8)
A/blue-winged Teal/Alberta/459/2017(H3N8)
A/mallard/Alaska/327/2013(H3N8)
A/northern pintail/Alaska/792/2013(H6N8)
A/swan/Shandong/W3701/2021(H11N2)
A/Wild Duck/South Korea/KNU2020-110/2020(H9N2)
A/emperor goose/Alaska/602/2012(H10N7)
Group4
Tree scale 0.005
H11N2 isolates in this study
H11N3 isolates in this study
H11N9 isolates in this study
Viruses isolates in YRD wetland, Shandong
Viruses isolates in Swan Lake wetland, Shandong
Bootstrap values
100
80
60
40
20
0

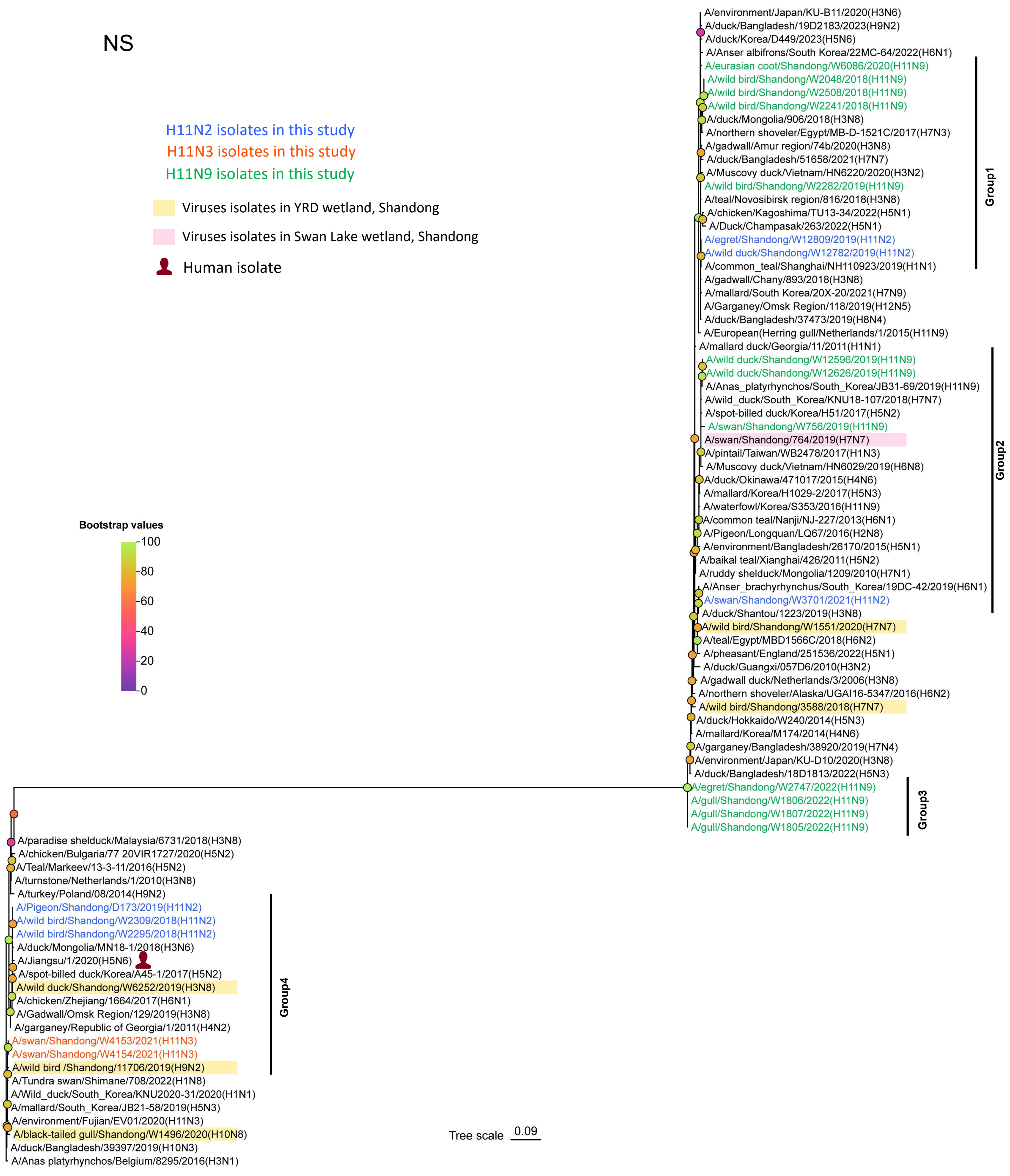
NS
H11N2 isolates in this study
H11N3 isolates in this study
H11N9 isolates in this study
Viruses isolates in YRD wetland, Shandong
Viruses isolates in Swan Lake wetland, Shandong
Human isolate
Bootstrap values
100
80
60
40
20
0
A/environment/Japan/KU-B11/2020(H3N6)
A/duck/Bangladesh/19D2183/2023(H9N2)
A/duck/Korea/D449/2023(H5N6)
A/Anser albifrons/South Korea/22MC-64/2022(H6N1)
A/eurasian coot/Shandong/W6086/2020(H11N9)
A/wild bird/Shandong/W2048/2018(H11N9)
A/wild bird/Shandong/W2508/2018(H11N9)
A/wild bird/Shandong/W2241/2018(H11N9)
A/duck/Mongolia/906/2018(H3N8)
A/northern shoveler/Egypt/MB-D-1521C/2017(H7N3)
A/gadwall/Amur region/74b/2020(H3N8)
A/duck/Bangladesh/51658/2021(H7N7)
A/Muscovy duck/Vietnam/HN6220/2020(H3N2)
A/wild bird/Shandong/W2282/2019(H11N9)
A/teal/Novosibirsk region/816/2018(H3N8)
A/chicken/Kagoshima/TU13-34/2022(H5N1)
A/Duck/Champasak/263/2022(H5N1)
A/egret/Shandong/W12809/2019(H11N2)
A/wild duck/Shandong/W12782/2019(H11N2)
A/common_teal/Shanghai/NH110923/2019(H1N1)
A/gadwall/Chany/893/2018(H3N8)
A/mallard/South Korea/20X-20/2021(H7N9)
A/Garganey/Omsk Region/118/2019(H12N5)
A/duck/Bangladesh/37473/2019(H8N4)
A/European(Herring gull/Netherlands/1/2015(H11N9)
Group1
A/mallard duck/Georgia/11/2011(H1N1)
A/wild duck/Shandong/W12596/2019(H11N9)
A/wild duck/Shandong/W12626/2019(H11N9)
A/Anas_platyrhynchos/South_Korea/JB31-69/2019(H11N9)
A/wild_duck/South_Korea/KNU18-107/2018(H7N7)
A/spot-billed duck/Korea/H51/2017(H5N2)
A/swan/Shandong/W756/2019(H11N9)
A/swan/Shandong/764/2019(H7N7)
A/pintail/Taiwan/WB2478/2017(H1N3)
A/Muscovy duck/Vietnam/HN6029/2019(H6N8)
A/duck/Okinawa/471017/2015(H4N6)
A/mallard/Korea/H1029-2/2017(H5N3)
A/waterfowl/Korea/S353/2016(H11N9)
A/common teal/Nanji/NJ-227/2013(H6N1)
A/Pigeon/Longquan/LQ67/2016(H2N8)
A/environment/Bangladesh/26170/2015(H5N1)
A/baikal teal/Xianghai/426/2011(H5N2)
A/ruddy shelduck/Mongolia/1209/2010(H7N1)
A/Anser_brachyrhynchus/South_Korea/19DC-42/2019(H6N1)
A/swan/Shandong/W3701/2021(H11N2)
Group2
A/duck/Shantou/1223/2019(H3N8)
A/wild bird/Shandong/W1551/2020(H7N7)
A/teal/Egypt/MBD1566C/2018(H6N2)
A/pheasant/England/251536/2022(H5N1)
A/duck/Guangxi/057D6/2010(H3N2)
A/gadwall duck/Netherlands/3/2006(H3N8)
A/northern shoveler/Alaska/UGAI16-5347/2016(H6N2)
A/wild bird/Shandong/3588/2018(H7N7)
A/duck/Hokkaido/W240/2014(H5N3)
A/mallard/Korea/M174/2014(H4N6)
A/garganey/Bangladesh/38920/2019(H7N4)
A/environment/Japan/KU-D10/2020(H3N8)
A/duck/Bangladesh/18D1813/2022(H5N3)
A/egret/Shandong/W2747/2022(H11N9)
A/gull/Shandong/W1806/2022(H11N9)
A/gull/Shandong/W1807/2022(H11N9)
A/gull/Shandong/W1805/2022(H11N9)
Group3
A/paradise shelduck/Malaysia/6731/2018(H3N8)
A/chicken/Bulgaria/77 20VIR1727/2020(H5N2)
A/Teal/Markeev/13-3-11/2016(H5N2)
A/turnstone/Netherlands/1/2010(H3N8)
A/turkey/Poland/08/2014(H9N2)
A/Pigeon/Shandong/D173/2019(H11N2)
A/wild bird/Shandong/W2309/2018(H11N2)
A/wild bird/Shandong/W2295/2018(H11N2)
A/duck/Mongolia/MN18-1/2018(H3N6)
A/Jiangsu/1/2020(H5N6)
A/spot-billed duck/Korea/A45-1/2017(H5N2)
A/wild duck/Shandong/W6252/2019(H3N8)
A/chicken/Zhejiang/1664/2017(H6N1)
A/Gadwall/Omsk Region/129/2019(H3N8)
A/garganey/Republic of Georgia/1/2011(H4N2)
A/swan/Shandong/W4153/2021(H11N3)
A/swan/Shandong/W4154/2021(H11N3)
A/wild bird /Shandong/11706/2019(H9N2)
Group4
A/Tundra swan/Shimane/708/2022(H1N8)
A/Wild_duck/South_Korea/KNU2020-31/2020(H1N1)
A/mallard/South_Korea/JB21-58/2019(H5N3)
A/environment/Fujian/EV01/2020(H11N3)
A/black-tailed gull/Shandong/W1496/2020(H10N8)
A/duck/Bangladesh/39397/2019(H10N3)
A/Anas platyrhynchos/Belgium/8295/2016(H3N1)
Tree scale 0.09

Figure S5

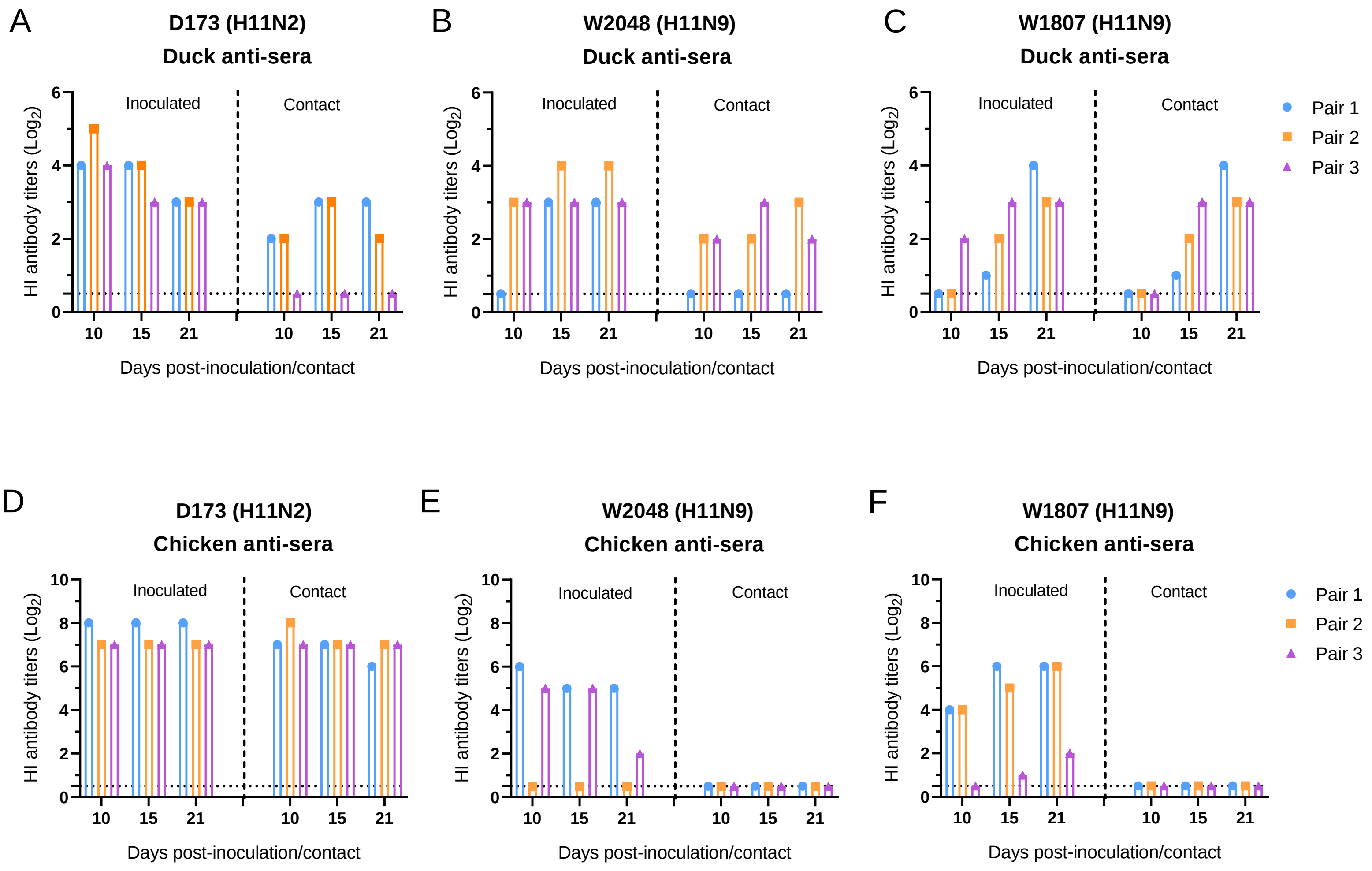
A
D173 (H11N2)
Duck anti-sera
B
W2048 (H11N9)
Duck anti-sera
C
W1807 (H11N9)
Duck anti-sera
D
D173 (H11N2)
Chicken anti-sera
E
W2048 (H11N9)
Chicken anti-sera
F
W1807 (H11N9)
Chicken anti-sera
Inoculated
Contact
HI antibody titers (Log2)
Days post-inoculation/contact
10
15
21
Pair 1
Pair 2
Pair 3
